# Modeling the United Oscillation and Wave of economic policy and urban planning employing spatial population dynamics

**DOI:** 10.1371/journal.pone.0305465

**Published:** 2024-07-17

**Authors:** Junghun Yang, Woorim Ko, Youngtae Cho

**Affiliations:** 1 Institute of Health & Environment, Seoul National University, Seoul, Korea; 2 Department of Public Health Science, Graduate School of Public Health, Seoul National University, Seoul, Korea; National University of Sciences and Technology, PAKISTAN

## Abstract

Modern urban dynamics are increasingly shaped by the interplay between economic policy and urban planning, yet often lack an integrated approach. This study bridges this gap by examining the dynamic equilibrium between these two realms using the “Oscillation and Wave Framework.” Specifically, we focus on the impact of variations in congestion parameter λ on urban sectoral spatial distribution and population dynamics. Our approach utilizes an advanced agent-based model to simulate interactions within an urban economic landscape, offering a detailed analysis of the relationship between agglomeration economies and congestion diseconomies. The results highlight the significant influence of congestion parameter adjustments on urban patterns, particularly in terms of cluster density and development. Therefore, this study not only provides a deeper understanding of the intricate balance between economic and urban planning factors but also emphasizes the necessity of incorporating these insights into urban planning and policy formulation for sustainable urban development. The findings also have important practical implications for addressing the dynamic complexities of urban environments, especially the interactions between different industries and their role in shaping urban structures.

## Introduction

### Background and objectives

Modern economic policy and urban planning face intrinsic issues stemming from the lack of an integrated approach [1]. For example, congestion caused by urban traffic often leads to the physical expansion of cities as well as implementation of policies that impose congestion costs on drivers, which can lead to a decrease in urban productivity due to job dispersion [2]. This gap between policies acts as a significant barrier to promoting urban agglomeration economies and managing the inefficiencies caused by congestion.

The integration between economic policy and urban planning is also essential for enhancing the vitality of urban spaces. To create livable communities, an integrated urban plan that considers various sectors, such as transportation, housing, employment, education, and social infrastructure, is necessary [3]. Moreover, enhancing urban planning through collaboration between planners and economic policymakers to strategically position suitable industries in industrial zones and city centers is crucial [4], as it will play a pivotal role in the formulation of comprehensive strategies for stimulating urban growth and preventing issues such as congestion. Therefore, the need for a feasible model linking these two aspects has become increasingly evident.

Existing studies related to the above situation can be divided into spatial economic models, spatial interaction models, and micro-simulation models. Spatial economic models [5–10] are primarily used for regional economy and land market analysis. These models use statistical methods and econometric approaches to explore various aspects of the urban economy. At their core, these models help understand and predict the interrelationship between economic activity and land use. However, although effective in analyzing the complexity of the urban economy, these models often rely on oversimplified assumptions and fail to sufficiently consider factors outside the economy.

Spatial interaction models [11, 12] play an important role in spatial planning and transportation. This model originates from the early work of Lowry [13] and analyzes the spatial interactions between individual actors within a city. The main advantage of this model is its ability to help understand the interactions between various elements within a city. However, this model has limitations in explaining the detailed behavior patterns of individual actors.

Finally, micro-simulation models [14, 15] focus on modeling the behavior of individual actors or household units, and are useful in the fields of spatial planning, transportation, and social policy. Micro-simulation helps analyze the impact of individual actors’ actions and decisions on urban systems [16]. However, these models require large amounts of data and complex calculations. Additionally, simplifications in the modeling process may not fully reflect the real complexities of human behavior.

In summary, economic policy has been treated as a spatial economic model; spatial planning has been treated as a spatial interaction model; and micro-simulation has been treated as a separate form; each emphasizes only one aspect—economic or spatial. To overcome the gap between policies that promote urban agglomeration economies and significantly impact congestion management, this study introduces an integrative approach—the “Oscillation and Wave Framework.”

“Oscillation” refers to the impact of economic policy on the market and the resulting volatility in economic activity through changes in the size of the workforce by industry sector. In other words, an understanding of oscillation is important for predicting and analyzing how the government’s economic policy will affect the labor market and how the number of workers will change in a specific industrial sector. These oscillations function as essential conceptual tools for coordinating economic flows and designing predictable development trajectories.

“Waves,” by contrast, refer to spatial changes and expansions that occur during urbanization, including worker mobility. They are crucial to understand the spatial continuity and trajectory of infrastructure development related to urban planning. Researchers analyze these waves to understand how workers move during the process of urbanization and how that movement affects the development and structure of urban space. In particular, mobility shapes the waves of urban space, which can inform the structural aspects of urban planning.

Through these concepts, the study’s “Oscillation and Wave Framework” helps understand the complex interaction between cities and the economy by systematically analyzing the interdependent relationship between economic policy and urban planning. The framework’s comprehensive overview of the impact of workforce changes and mobility on the development and transformation of urban spaces provides a fundamental understanding for sustainable urban development and effective economic policymaking. It aims to make an important academic contribution that theoretically clarifies the complex relationship between urbanization processes and economic oscillations. In practice, it enables the effective coordination and prediction of urban planning and economic policies.

The purpose of this integrated approach is to analytically integrate the complex interactions between economic policy and urban planning to comprehend the structure through which changes in the size of the labor force drive market oscillations, and how labor mobility shapes waves in urban spaces. This study aims to comprehensively understand how unpredictable economic volatility and the expansion of urban space influence each other, and to develop effective coordination and forecasting strategies based on this association. This study seeks to provide a theoretical and practical framework that is essential for understanding and managing the interaction of unpredictable volatility and spatial expansion in the fields of economics and urban planning [17, 18].

### Theoretical framework

The “Oscillation and Wave Framework” is an innovative approach to understanding the complex interaction of economics and urban planning. The origins of this framework can be traced to a variety of academic disciplines, with particular influences from economic geography [19–21] and complexity science [22, 23]. Previous studies have mainly focused on individual phenomena, such as the inherent oscillations of economic systems or the expansion of urban space. However, the “Oscillation and Wave Framework” provides new insights by modeling the interaction of these two phenomena.

We now review the literature based on the Oscillation and Wave Framework and divide existing studies in three research streams: urban economic oscillations, urban spatial waves, and integrated simulation development.

First, research on urban economic oscillations can be categorized into two aspects: population dynamics and economic systems. May [24] introduced a fundamental concept in understanding population dynamics through a mathematical equation. This equation illustrates the dynamic changes in population based on the parameters that influence growth rates and limitations. It demonstrates how different values of these parameters can lead to a variety of population states, from stable to oscillatory, and even chaos. It is thus instrumental in comprehending the complex interplay between population growth and environmental constraints. These dynamic changes provide significant insights into the complex interactions between population dynamics and economic systems [25]. Turchin [26] explored periodic change patterns in population dynamics using logistic and Lotka–Volterra models to analyze the impacts of environmental carrying capacity and interactions between predators and prey [23, 27, 28]. Although these studies provide a profound understanding of the intrinsic causes of economic systems, they are limited in fully encompassing temporal and spatial changes and complex interactions. Complex system economics views oscillations occurring naturally within economic systems as emergent phenomena originating from the interactions between economic actors and their environments [29]. However, these intrinsic dynamics pose challenges in explaining the processes that create complex patterns, making predictions more difficult [30]. Therefore, this study addresses these issues by integrating the concepts of oscillations and waves to systematically analyze the interactions between economy and space.

Second, our review of prior research on “waves” focuses on the centrifugal and centripetal forces that spatially expand the interactions within the economy. According to Krugman [21], economic space is formed by the interaction between the centripetal force, wherein manufacturers concentrate production in areas that allow the rapid procurement of intermediates, and the centrifugal force, which emanates from immobile peasants. The balance between these forces determines the spatial distribution of the economy. Krugman [21] conceptualized the distribution of manufacturing as the sum of sine waves with different wavelengths, explaining that each temporal passive has a unique growth rate and determines spatial patterns, thereby facilitating self-organizing processes in the economy. Fujita et al. [19] demonstrated that the spatial concentration of economic activities is closely linked to agglomeration economies. This creates an environment that induces continuous concentration, owing to factors such as the role of industrial clusters, presence of cities, and differences between industrial and agricultural areas. Krugman [21] and Fujita et al. [19] modeled the interaction of centripetal and centrifugal forces that constitute economic spaces and addressed the self-organizing process of cities; however, these studies are limited in fully capturing the multi-layered economic structure of urban areas.

Batty [31], van Wissen [32], and Yang and Ettema [1] contributed to urban planning and economic policy formulation using advanced urban economic models. Batty [31] introduced a magnetism concept similar to Krugman’s [21] centripetal force, modeling the balance between centripetal and centrifugal forces using a distance decay equation. This approach emphasizes economies of scale by dividing the structure of urban systems into potential, population, and development; however, it reveals limitations in comprehensively capturing the complex interactions of the actual urban environment. van Wissen’s [32] SimFirms model simulates how firms’ spatial distribution and movement contribute to macroeconomic patterns and waves. The model identifies the micro-interactions of economic activities by considering the characteristics of each firm. Yang and Ettema [1] explored how spatial externalities affect the spatial distribution of economic activities through modeling. However, these studies have certain limitations in meticulously modeling the dynamics at the individual firm level while fully assessing the impact of these changes on the overall urban economy. This suggests the need for a deeper approach to understand and predict the overall economic situation of a city in urban planning and economic policy decisions.

The research directions of this study derived from the above literature review are as follows. First, this study proposes analyzing the trade-off between “agglomeration economies” and “congestion,” positioning it as a key element in modeling urban growth and decline [33]. As economic policies are linked to agglomeration economies, whereas urban planning is associated with congestion inefficiencies, our study models the selection and interaction of various economic activities within a city by converting the advantages of agglomeration economies and inefficiencies due to congestion into potential functions through the economic landscape [1].

Second, this study develops a new simulation model that links market oscillations and urban spatial waves [34–36] using the concept of potential. The model aims to simulate the economic and spatial dynamics occurring within cities, predict the spatial expansion in urbanization and infrastructure development, and simultaneously capture the vitality of the labor market and economic oscillations.

Third, by setting individual workers as the basic unit of analysis, this study reflects the contemporary societal trend in which the focus of employment shifts from firms to cities [37]. As a collective of workers from various industrial sectors, the city is viewed as a major driver shaping its form and function through economic activities and movement patterns. This study models the distribution of workers within a city and the interactions among industrial sectors for a new understanding of the city’s economic context.

These three main directions are the strategic objectives of this study, with the aim of contributing to academic research and providing practical applications in the fields of economic policy and urban planning. This study thus provides theoretical models and quantitative guidelines that contribute to the integrated development of urban planning and economic policy. This is expected to play a significant role in effectively understanding and predicting complex phenomena in cities.

## Materials and methods

### Study design

This study developed an experimental research method using an agent-based model to understand the interaction between urban agglomeration economies and the congestion diseconomy. The first phase, the “Oscillations and Waves in Simple Parameters” experiment, analyzed the quantitative mechanisms of initial congestion state formation and focused on the measurements of key indicators, such as population, births, development activity and Cluster Density Index (CDI). The second phase, the “Oscillation and Waves in Complex Parameters” experiment, simulated the life cycle of a city, identifying patterns of urban growth and decline and highlighting potential growth prospects for traditional, innovative and service industries.

The experimental space was set up with 2,500 cells organized into a 50 × 50 grid, with each cell representing a potential location of the agent. In the initial setting, a total of 150 worker agents were assigned, 50 each from traditional, innovative, and service sectors. They served as basic units to simulate economic interactions and the dynamic distribution of population in an urban space. The degree of clustering between agents was determined by CDI based on a distance variable value of 10, which was used as one of the key dynamics of the simulation. The first and second phases of the experiment included 340 and 740 time steps, respectively, ensuring sufficient time scale to observe long-term urban patterns and changes.

Additionally, this study conducted sensitivity analysis to verify the model. This analysis assessed the impact of each scenario on model output by applying changes to single and composite potential weights for each scenario. This method precisely analyzed how each variable affected urban patterns and economic activities and confirmed the model’s robustness. In particular, this approach can be used as a methodology that can be directly applied to urban planning and economic policy.

The application of this research design and results is expected to provide practical and specific directions for future urban planning and economic policy formulation; additionally, it hopes to make an important contribution to resolving the complexity of the urbanization process. In this way, this study seeks to provide an effective tool to better understand and predict economic and spatial changes in cities.

### Operational framework

This study examines the complex interrelationships between the advantages of urban agglomeration economies and the economic losses due to congestion, being grounded in spatial population dynamics. Crucially, it also defines these interactions through the economic landscape and develops a multi-level framework that utilizes a cell-based structure to model the dynamic interactions of population groups [38]. In this framework, a macro-level analysis of the economic landscape clearly delineates the relationship between the development potential and congestion inefficiencies, applying it to the meso-level cell structure to simulate the changes in population groups. The birth process symbolizes the employment creation of businesses, integrating modeling techniques that finely tune complex phenomena such as the growth, death, and relocation of population groups at the micro level.

**Fig 1:**
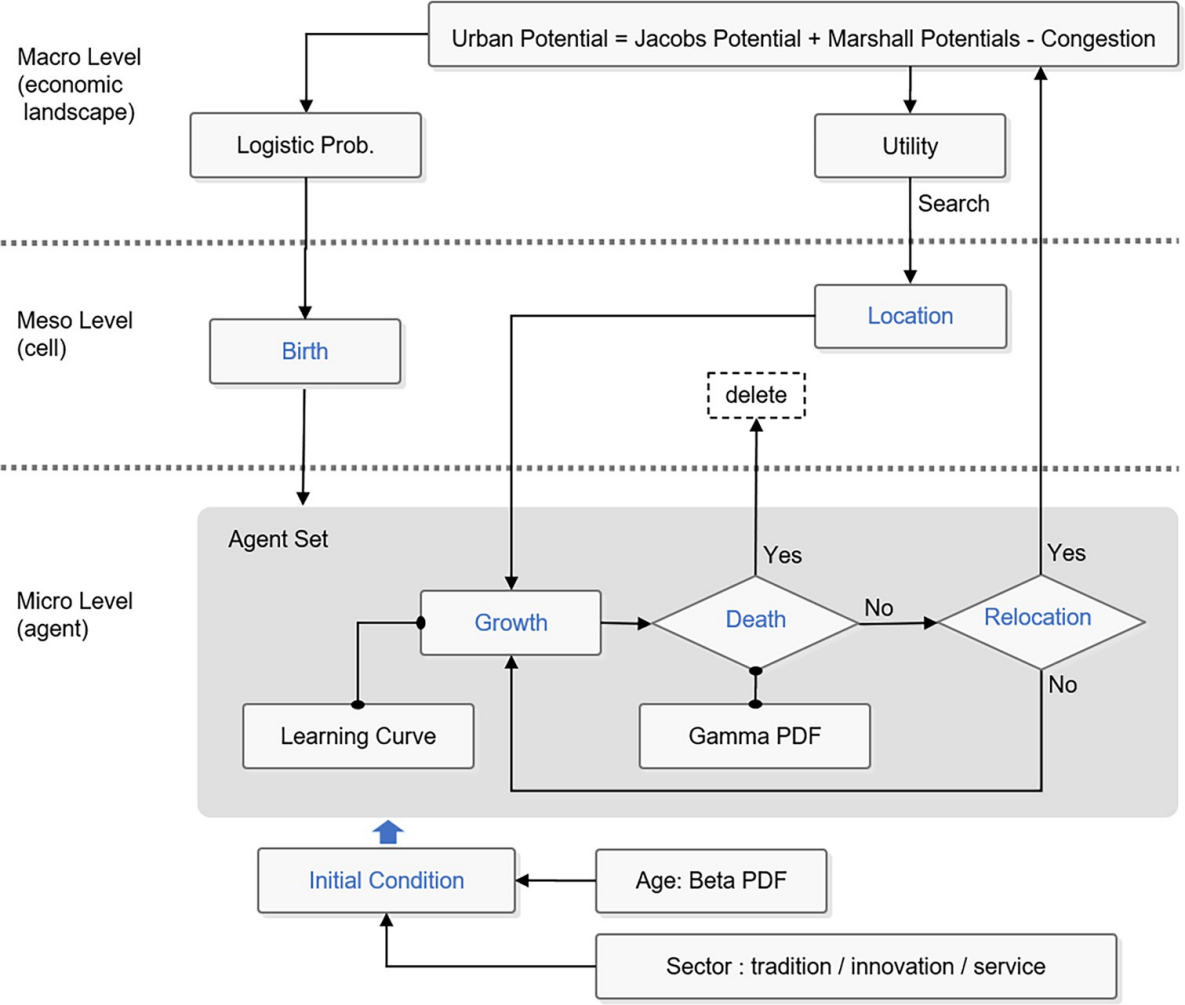
Framework.

This model emphasizes quantifying the economic potential of cities and enabling population groups to determine their optimal locations based on utility [39]. The agent-based modeling and simulation methodology [40, 41] offers flexibility in quantitatively analyzing urban oscillations, including complex changes in urban spaces and characteristics of population groups. Thus, this framework is a vital tool for systematically understanding how the micro decisions of individual populations affect the overall structure and function of a city. As such, this study contributes to a deeper understanding of the complex characteristics and dynamics of urban systems through various scenario analyses. Fig 1 visually summarizes the structural approach of the study, as well as its theoretical and methodological foundations.

The agent-based model (ABM) used in this study is designed to sophisticatedly capture the interactions between populations and businesses within urban economic landscapes. The model is based on the heterogeneity, interactions, and adaptability of individual actors within a city, simulating how these actors influence each other and contribute to the formation and change of urban patterns. The model includes the following key assumptions and parameters.

The main assumptions of the model include actor heterogeneity, interactions, and adaptations. First, all actors have attributes, such as industry sector and age, as well as related preferences. This affects their mobility and employment. Second, actors directly or indirectly influence each other, creating patterns such as urban cluster formation, sprawl development, and congestion phenomena. Third, depending on the outcome of their interactions with their surroundings, actors modify their behavior.

The following are the main parameters of the model. The congestion parameter λ represents the degree of congestion within the city, which changes according to the city’s congestion potential. Urban Potential (UP) is an indicator that measures the economic attractiveness of a specific area within a city and plays an important role in the actors’ selection of location. Movement decision rules are used by actors to make decisions about moving within a city or migrating to a new location. Learning and development models are parameters that model the experience of actors and their growth over time, which includes increased individual productivity and job skills development.

The following methods are used to capture interactions within the urban economic landscape. ABM divides urban space into various cells, each of which has a specific UP. Actors determine optimal locations based on this potential, and the resulting population distribution and economic activity patterns indicate the formation of urban clusters, sprawl, and congestion. Actors’ mobility decisions also serve as a key factor in driving interactions and economic dynamics within cities. For example, actors located near specific industrial clusters may earn higher incomes or have better employment opportunities due to the economic potential of the area. These dynamic interactions drive economic and spatial changes in cities, providing a basis for policymakers and urban planners to construct more effective urban development strategies.

Through the application of models, this study analyzes complex economic and demographic interactions in an urban space at a micro level, thereby helping policymakers and urban planners better understand and predict economic and spatial changes in cities.

### Model description

#### Potential model

Four primary types of functions are recognized in the field of distance decay functions. The reciprocal and negative exponential functions demonstrate a marked decrease as they approach the origin, whereas the step function exhibits a sudden change beyond the critical distance. Conversely, the Gaussian function exhibits a more gradual initial decay [42]. These characteristics are advantageous for modeling that delineates the economic interactions between a city’s core and periphery; they are also well suited for generating varied configurations of economic potential by adjusting parameters. This study employs the Gaussian decay function to analyze the changes in the spatial range of the potential influence. A higher σ value indicates a gradual decrease in potential influence across a broad area, whereas a lower σ value signifies a rapid decline within a confined range.

The distribution of urban potential (UP) emerges from the integration of the Jacobs potential (JP), Marshall potential (MP), and congestion diseconomies [1], and plays a pivotal role in analyzing the formation of economic interaction hubs within the city. The overlapping Gaussian functions with varying σ values create optimal points of potential within the urban landscape. In this study, the JP is defined to quantify the agglomeration of heterogeneous industrial sectors at location *i* as follows:

$$JP_{i}=\underset{j}{\sum }N_{j}\;\exp(\frac{-{\left(d_{ij}\right)}^{2}}{2\sigma^{2}})$$ (1)

where *JP*_*i*_ represents the JP at location *i*, *N*_*j*_ denotes the total number of workers at location *j*, *d*_*ij*_ signifies the distance between locations *i* and *j*, and σ is the parameter indicating the spatial range of influence of the potential. This formula is essential for understanding the spatial distribution of urban economic activity. The MP model in this study is grounded in Alfred Marshall’s economic theories [43, 44] and quantitatively analyzes the external economic effects of industrial agglomeration, such as information exchange, technology diffusion, and a shared labor force. This model is defined using the following formula:

$$MP_{is}=\underset{j}{\sum }N_{js}\;\exp(\frac{-{\left(d_{ij}\right)}^{2}}{2{\sigma_{s}}^{2}})$$ (2)

where *MP*_*is*_ represents the MP at location *i* for sector *s*, *N*_*js*_ indicates the number of workers in sector *s* at location *j*, *d*_*ij*_ denotes the distance between locations *i* and *j*, and *σ*_*s*_ is the parameter controlling the spatial range of influence for the sector-specific potential. This model quantitatively assesses the spatial agglomeration of specific industries and the resultant economic impact on surrounding areas, thus providing crucial insights for regional economic growth strategies. The *σ* values serve as critical indicators of the extent to which economic impacts can proliferate, offering foundational data essential for modeling various scenarios related to the agglomeration of economic activities within a region. As economic activities concentrate in a city, congestion diseconomies arise, leading to negative impacts on urban functionality such as increased traffic congestion, environmental pollution, and rising housing costs. To quantitatively understand these congestion diseconomies, the following model is introduced:

$${\mathrm{CON}}_{i}=\underset{j}{\sum }N_{j}\;\exp(-\lambda \cdot d_{ij})$$ (3)

where *CON*_*i*_ represents the level of congestion diseconomies at location *i*, *N*_*j*_ denotes the total number of workers at location *j*, *d*_*ij*_ signifies the distance between locations *i* and *j*, and *λ* is the parameter adjusting the rate of decrease in congestion diseconomies. This model mathematically expresses how the impact of congestion diseconomies diminishes as distance increases. Our study combines the JP and MP with congestion diseconomies to extract the UP. We conducted a thorough analysis of the interplay between economic agglomeration and congestion diseconomies in urban settings. The quantitative model of the UP is as follows:

$$UP=\beta_{1}JP+\beta_{2}MP_{\mathrm{tras}}+\beta_{3}MP_{\mathrm{innos}}+\beta_{4}MP_{\mathrm{service}}-\beta_{5}CON$$ (4)

where *UP* represents the Urban Potential, whereas JP, *MP*_*tras*_, *MP*_*innos*_, and *MP*_*service*_ signify the MPs of the traditional, innovative, and service industries. *CON* denotes the congestion diseconomies and the *β* coefficients adjust the relative significance of each potential. This model offers essential insights for urban planning and economic development strategy formulation by analyzing the spatial patterns in urban economics and the variations in economic agglomeration benefits and congestion diseconomies with distance from the city center.

**Fig 2:**
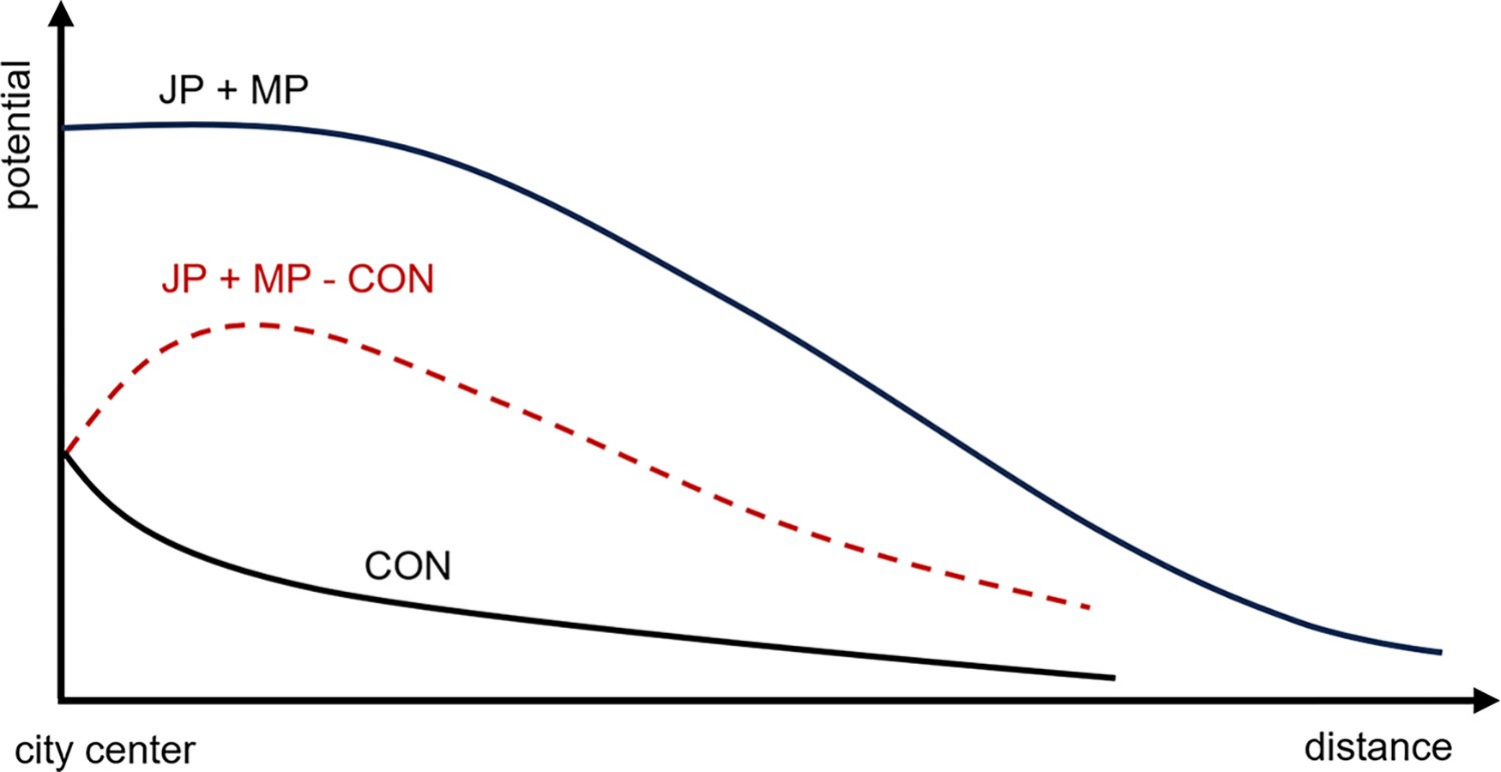
Potential landscape.

The “potential landscape” graph (Fig 2) shows the changes in the potential of urban economic activities, illustrating the diminishing economic potential with increasing distance from the city center. The summation of the JP and MP peaks in the city center symbolizes the point where the benefits of economic agglomeration are maximized. Conversely, the CON curve, which indicates the negative impact of congestion diseconomies on economic potential, is positioned below the sum of JP and MP, suggesting economic losses induced by congestion. These data provide critical insights for economists and urban planners in balancing the interplay between economic agglomeration and congestion and in devising policies to optimize urban economic potential while minimizing congestion-related issues.

Table 1 shows an overview of the parameter settings that constitute the UP model. In this table, the *σ* value for JP is set at 3.3, implying that JP can exert influence over a comparatively broader area relative to other potentials. Conversely, the *σ* value for the service industry MP (*MP*_*service*_) is 1.7, suggesting that this potential represents more concentrated economic interactions. The *β* coefficients play a pivotal role in adjusting the impact of each potential in the calculation of UP, and they are meticulously determined based on theoretical and empirical data. The weights assigned to *UP*_*tras*_, *UP*_*innos*_, and *UP*_*service*_ reflect the relative importance of each potential impact on the urban economy. These weights are used to analyze the influence of specific potentials, either doubling or halving their effects, within certain sectors of the urban industry. Such weight configurations are crucial for understanding the operational principles of each potential and for providing vital information to policymakers in urban planning and economic policy formulation.

**Table 1:** Potential parameter set.

Potential	JP	MP_tras_	MP_innos_	MP_service_	CON (λ)
σ	3.3	2.7	2.2	1.7	0.9
UP	*β*_*1*_	*β*_*2*_	*β*_*3*_	*β*_*4*_	*β*_*5*_
UP_tras_	1	0.5	0.5	0.5	1
UP_innos_	1	0.5	0.9	0.5	1
UP_service_	1	0.5	0.5	0.5	1

#### Birth model

The birth model employs a logistic regression function to predict the probability of new economic activities based on UP, denoted as *u*. This study represents the probability of the emergence of one new worker. This model is defined as follows:

$$\mathrm{Birth}(u)=\{\begin{array}{ll} \frac{1}{1+\exp(-(cu-d)/S)}, & u<\xi_{\mathrm{birth}}\\ 0, & u\geq \xi_{\mathrm{birth}} \end{array}$$ (5)

**Fig 3:**
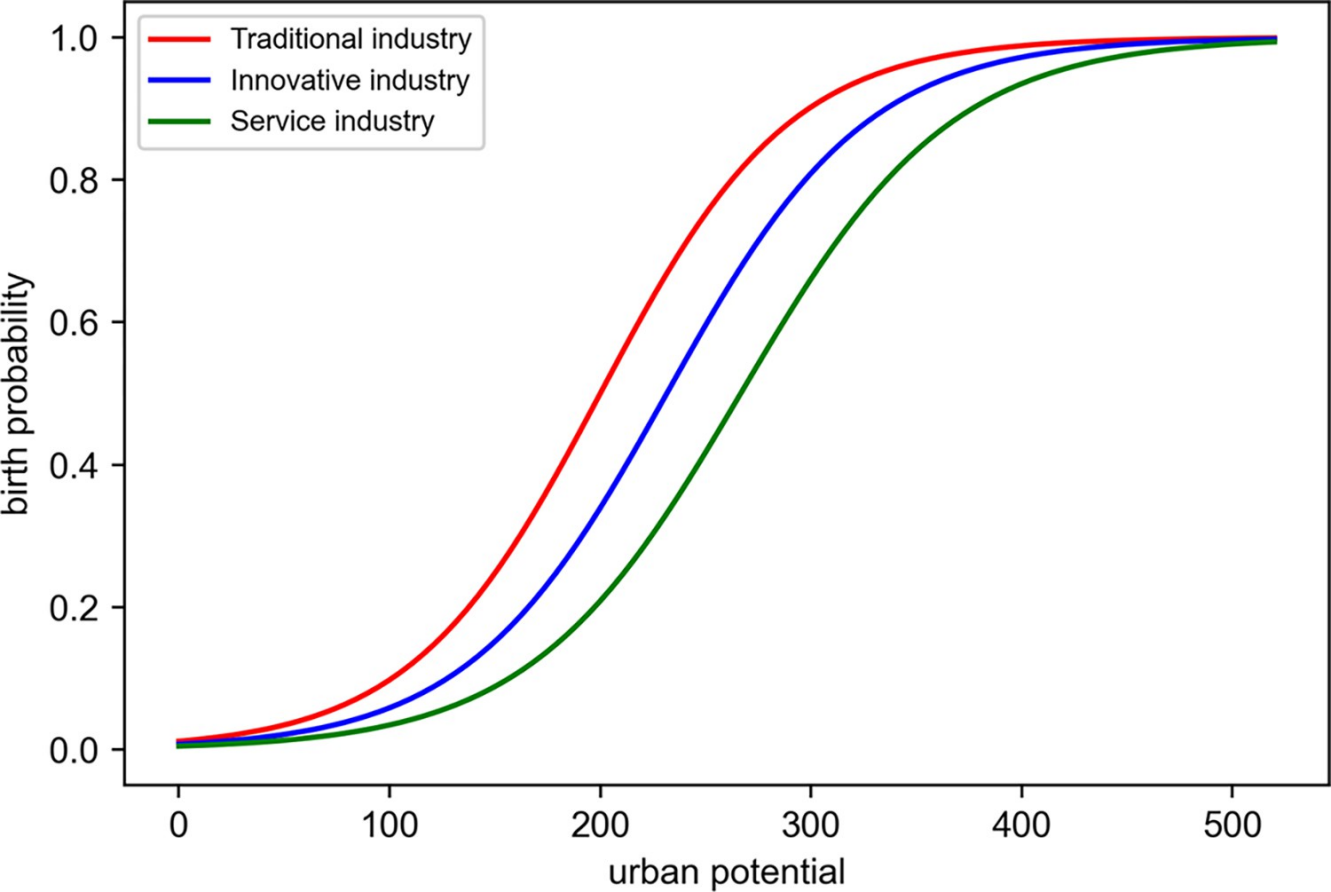
Birth probability in urban potential.

The above function modulates the emergence of economic activities through the interaction between UP and sector-specific control variables (*c*, *d*, and *S*). It intricately sets the inception point of economic activities for each sector using a threshold value, *ξ*_*birth*_, beyond which no economic activity occurs at a certain UP level. Fig 3 shows the trends in the probability of birth for each economic sector as UP increases. The traditional, innovative, and service industries show different rates of increase in birth probability with an increase in UP, and a saturation point can be observed at specific UP levels. This graph demonstrates how the likelihood of economic activity responds to changes in UP, providing strategic decision-makers in urban planning and economic policy with a vital analytical tool.

Table 2 presents the “birth parameter set,” which systematizes key parameters that quantify the dynamics of new activities in various economic sectors. In traditional industries, the *c* value is fixed at 1, indicating a consistent response to even subtle UP changes. This was interpreted with a *d* value of 200, signifying a 50% probability of activity occurrence at this potential, whereas the *S* value, which adjusts the rate of change, was set at 45. The *ξ*_*birth*_ is established at 400, suggesting that new economic activities are restrained when UP exceeds this figure. For innovative industries, the *c* value is adjusted to 0.95, reducing the sensitivity to responses. The *d* value is presumed at 220, reaching a 50% activity occurrence probability at this UP, with the *ξ*_*birth*_ threshold set at 450, indicating the initiation of economic activities at a higher potential than traditional industries. The service sector sets the *c* value at 0.9, reflecting the most gradual response to potential changes, with the potential threshold for activation, *b*, at 240, and *ξ*_*birth*_ at 500, having the highest values. These parameters establish a scientific basis for determining the growth prospects and policy directions in each economic sector, providing vital information for sector-specific growth strategies and policy decisions. The initial age range of the agents generated by the birth model was set to 20–30 years, mirroring the age distribution of the economically active population and contributing to the dynamics of the model.

**Table 2:** Birth parameter set.

Sector	c	d	S	*ξ_birth_*
Traditional Ind.	1	200	45	400
Innovative Ind.	0.95	220	45	450
Service Ind.	0.9	240	45	500

#### Learning model

The learning model simulates the individual productivity growth based on work experience, denoted by *w* [45, 46]. This model is composed of a combination of logarithmic and decay functions and considers both the effects of learning from experience and the decline in skills over time. The formula for the model is as follows:

$$\mathrm{Learning}(w)=\{\begin{array}{ll} k\cdot \tau_{s}\cdot \log(1+(\mu_{s}\cdot w)), & w\leq \xi_{\mathrm{learning}}\\ k\cdot \tau_{s}\cdot \log(1+(\mu_{s}\cdot w))-\eta \cdot r\cdot {\left(x-\xi \right)}^{2}, & w>\xi_{\mathrm{learning}} \end{array}$$ (6)

The learning model incorporates key parameters *κ*, *τ*, *μ*, *η*, and *r*, representing control coefficients, initial productivity per sector, level of learning, another set of control coefficients, and the decay rate, respectively. Notably, *ξ*_*learning*_ signifies a critical threshold where the productivity increase undergoes a transformation. According to this model, as work experience increases, productivity initially grows following a logarithmic function; however, once it surpasses a specific threshold, *ξ*_*learning*_, the rate of the increase begins to decline. This finding indicates a diminishing effect of learning with prolonged job performance and advancing age, illustrating the limitations of productivity growth. The parameter set in the learning model (Table 3) details the sector-specific parameters required for modeling productivity growth based on experience. Parameters *κ*, *τ*, *μ*, *η*, and *r* for each sector reflect the initial magnitude of productivity increase due to experience and the tendency for skill decay.

**Table 3:** Parameter sets in the learning model.

Sector	*κ*	*τ*	*μ*	*η*	*r*	*ξ_learning_*
Traditional Ind.	0.01	50	1.5	0.001	1.0	20
Innovative Ind.		80	0.5		1.7	
Service Ind.		60	1.0		1.3	

**Fig 4:**
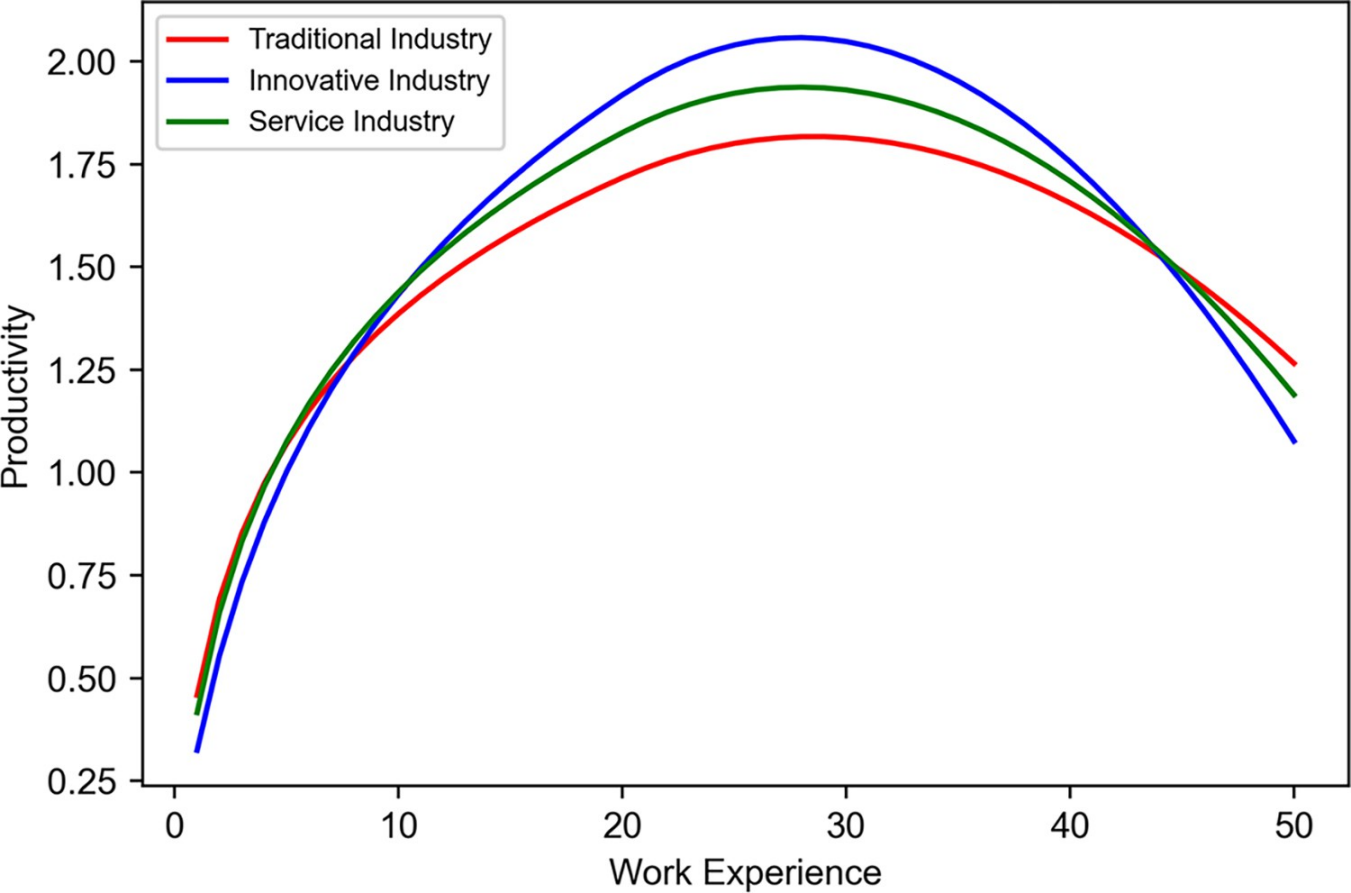
Learning curve of work experience.

The learning curve of work experience (Fig 4) allows for a visual comparison of how productivity in traditional, innovative, and service industries changes with increasing experience. This graph illustrates a rapid increase in productivity at the onset of experience but a deceleration in the rate of growth upon exceeding certain levels of experience. This is thus a crucial tool for demonstrating the limits of productivity resulting from accumulated experience and provides essential analytical data for the development of human resource strategies across different sectors.

#### Death model

In estimating the probability of employment termination for workers, the death model utilizes the Gamma distribution probability density function, with working period *h* as a key variable. This model is defined by shape parameter *ω* and scale parameter *θ* and is normalized by the Gamma function *Γ(ω)*. This model is represented by the following formula:

$$\mathrm{Death}(h)=h^{\omega -1}\frac{e^{-h/\theta }}{\theta^{\omega }\cdot \Gamma (\omega )}$$ (7)

**Fig 5:**
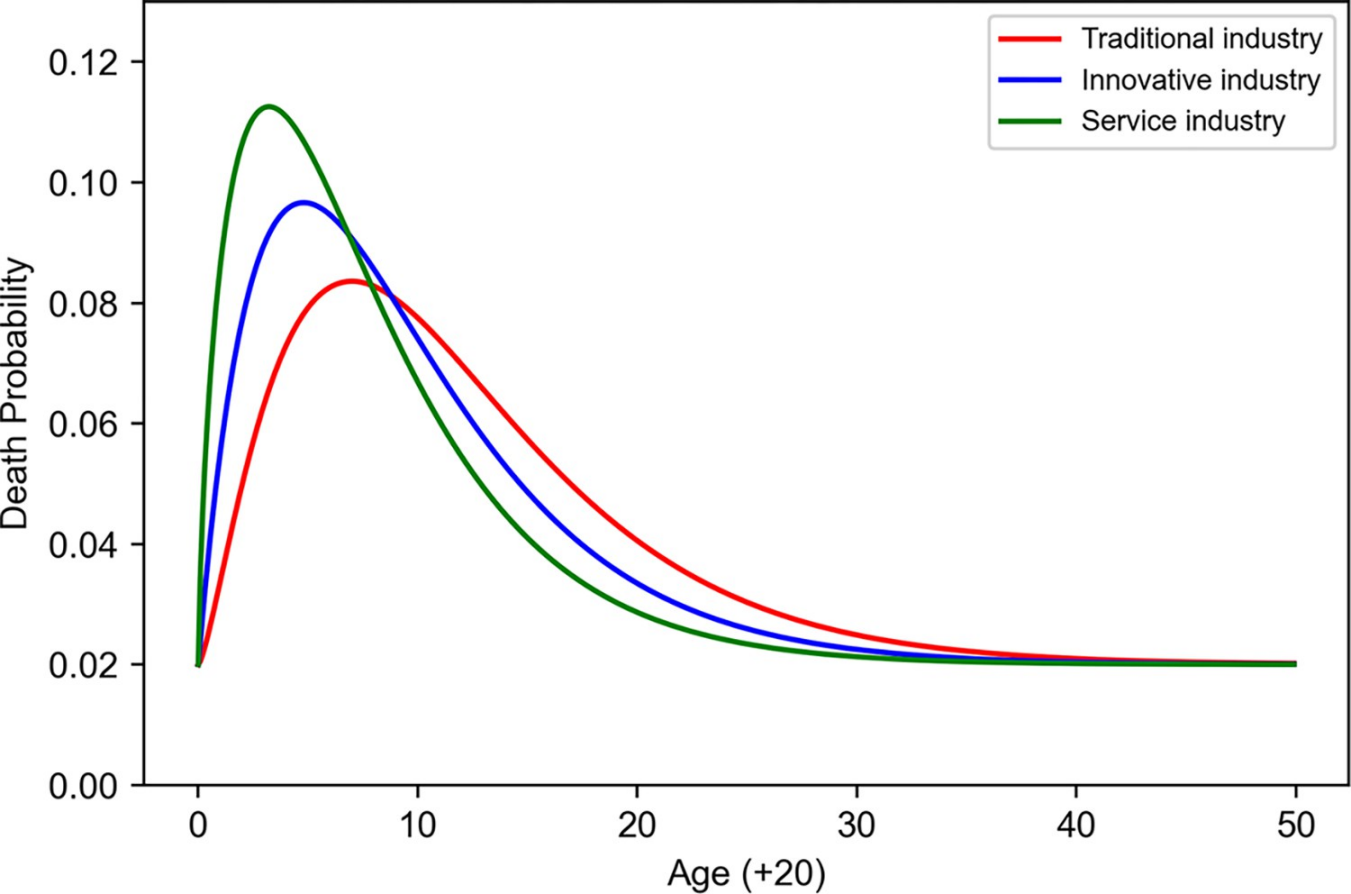
Probability of death in each age group.

This serves as an essential tool for predicting the timing of employment termination in specific industries. This reveals that traditional industries display a long-tail distribution, innovative industries exhibit a wide range of retirement probability distributions, and the service sector shows the most broadly dispersed distribution. In traditional industries, the scale parameter *θ* is set at 5.0, in innovative industries at 4.8, and in the service sector at 4.6, reflecting how each industry differs in the temporal scale of worker retirement (Table 4). The graph depicting the probability of employment termination by age (Fig 5) visually demonstrates how retirement probabilities vary with age across industries. In all sectors, the probability of employment termination peaks at a certain age range and then decreases, serving as a vital indicator of workforce management and strategic planning.

**Table 4:** Parameter set in the death model.

Sector	*ω*	*θ*	±*y*
Traditional Ind.	2.4	5.0	0.05
Innovative Ind.	2.0	4.8	0.05
Service Ind.	1.7	4.6	0.05

#### Relocation rule

The relocation rule models the mobility decisions of workers. This rule assigns probabilities to three choices: remaining in the current city, moving to another city, or relocating to a new, unoccupied area (Table 5).

**Table 5:** Relocation rule.

Mobility Decision	Probability
Stay	0.9
Move to existing city	0.09
Move to empty area	0.01

This rule quantifies the likelihood of each mobility option, guiding workers’ decisions based on their UP and utility. The process of making relocation decisions is inherently complex, and this rule simplifies and models the process. In this study, workers selected their movements based on the given probabilities, drawing on the theories of Simon [47] and Krugman [21], whose research provided profound insights into population mobility, contributing to our understanding of the spatial distribution of economic activities and regional mobility [1].

#### Location model

The location model offers a utility-based approach for quantifying the factors influencing locational decisions. In this model, the utility function represents an individual’s preference for a given area’s UP and is expressed by the following formula:

$$\mathrm{Location}(u_{i})=\frac{\exp(u_{i})}{\sum_{j}\exp(u_{i}\;\exp(\frac{-{\left(d_{ij}\right)}^{2}}{2{\delta_{s}}^{2}}))}$$ (8)

*u*_*i*_ represents the UP of area *i*, *σ*_*s*_ denotes the sector-specific distance-decay parameter, and *d*_*ij*_ indicates the distance between areas *i* and *j*. The utility function forms a normalized probability distribution that reflects the relative probability of an individual choosing area *i*. The probability of selecting area *i* increases as its UP increases and as its distance from other areas decreases.

The model’s core, the normalized probability distribution, demonstrates that the likelihood of choosing a particular area increases with higher UP and proximity to other areas. Our approach, which does not consider relocation barriers in the numerator, ensures that the UP of each area is preserved in its purest form, thereby directly influencing the probability of the choice. Conversely, the normalization process includes relocation barriers in the denominator, which accounts for the overall difficulty and cost of moving decisions. This allows areas farther away to be considered for selection if their utility is sufficiently high to overcome barriers to movement.

**Fig 6:**
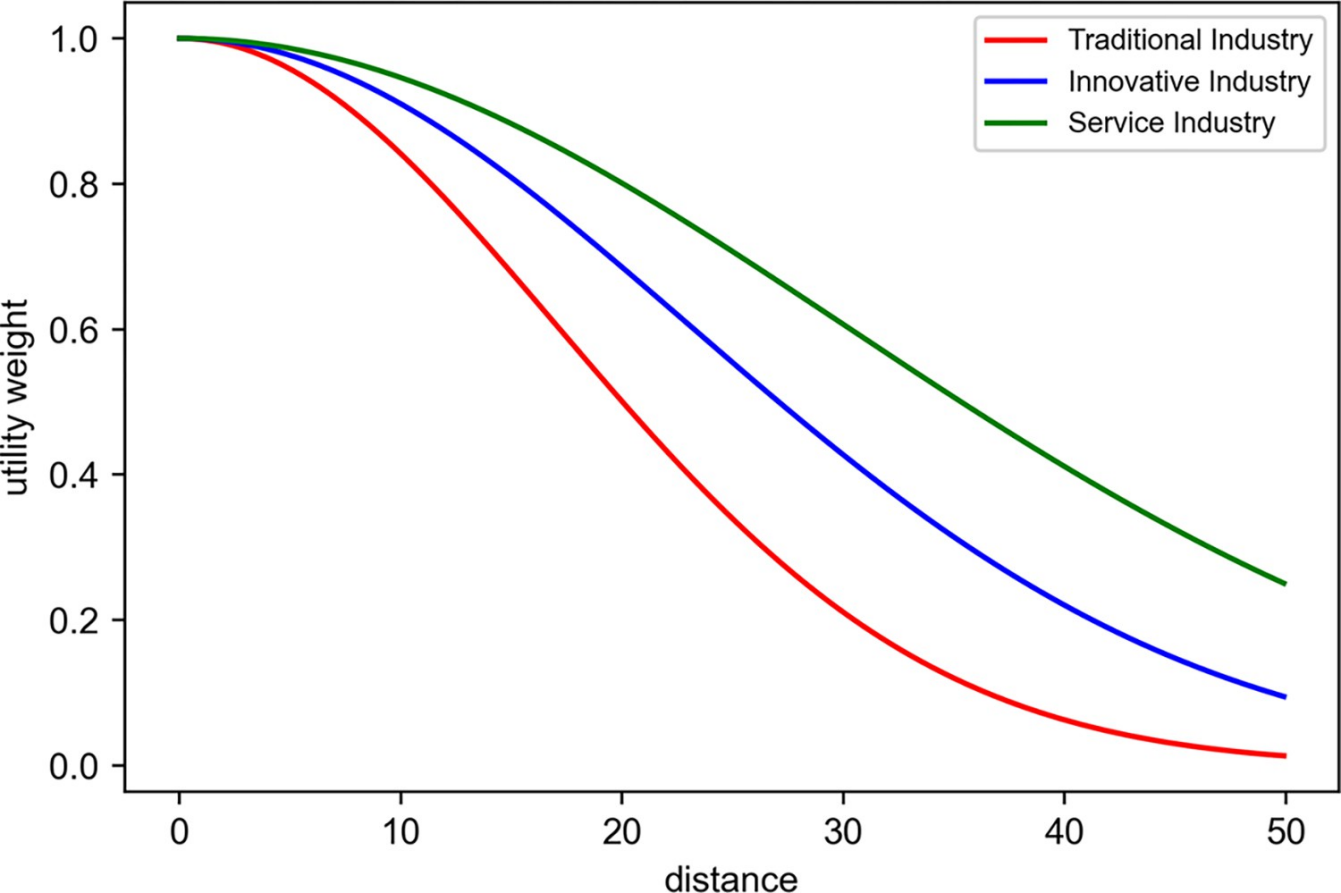
Utility weight for each distance.

Table 6 illustrates sector-specific distance-decay parameters *δ*_*s*_, a measure of how utility decreases with increasing distance, suggesting that each sector evaluates the attractiveness of geographic locations differently. Fig 6 illustrates the utility reduction trends based on these parameters, with traditional industries showing the highest constraints on movement and locational decisions.

**Table 6:** Parameter set in the location model.

Sector	*δ_s_*
Traditional Ind.	17
Innovative Ind.	23
Service Ind.	30

#### Age distribution model

Our age distribution model played a crucial role in setting the age structure of the initial population data [48]. This model, which determines the population ratio by age, is defined through parameters *m*, *n*, and *φ*, which modulate the shape and range of the age distribution. The formula for this model is as follows:

$$\mathrm{Age}(x)=\frac{\left(1/\varphi \right)}{B(m,n)}{\left(x\right)}^{m-1}{\left(1-x/\varphi \right)}^{n-1}$$ (9)

*x* represents age, while *m* and *n* are parameters that determine the shape of the age distribution, and *φ* denotes the range of this distribution. The Beta function, *B(m*, *n)*, ensures a uniform application across the entire population. As shown in Table 7, the parameter settings reveal the distinctive age distribution characteristics of each economic sector. For the traditional industry, *m* = 5.0 and *n* = 4.4 illustrate a broad age distribution spanning younger to middle-aged groups, reflecting the sector’s need for a diverse workforce age range. The innovative industry, set at *m* = 4.0 and *n* = 5.0, indicates a higher proportion of younger individuals, hinting at the potential for creative and innovative activities to be driven by younger talent. The service industry, with *m* = 4.5 and *n* = 4.8, displays a relatively balanced age distribution, suggesting a stable workforce encompassing various age groups. The consistent setting of *φ* = 80 across all sectors signifies that the age distribution is maintained uniformly.

**Table 7:** Parameter set in the age distribution model.

Sector	m	n	*ϕ*
Traditional Ind.	5.0	4.4	80
Innovative Ind.	4.0	5.0	80
Service Ind.	4.5	4.8	80

**Fig 7:**
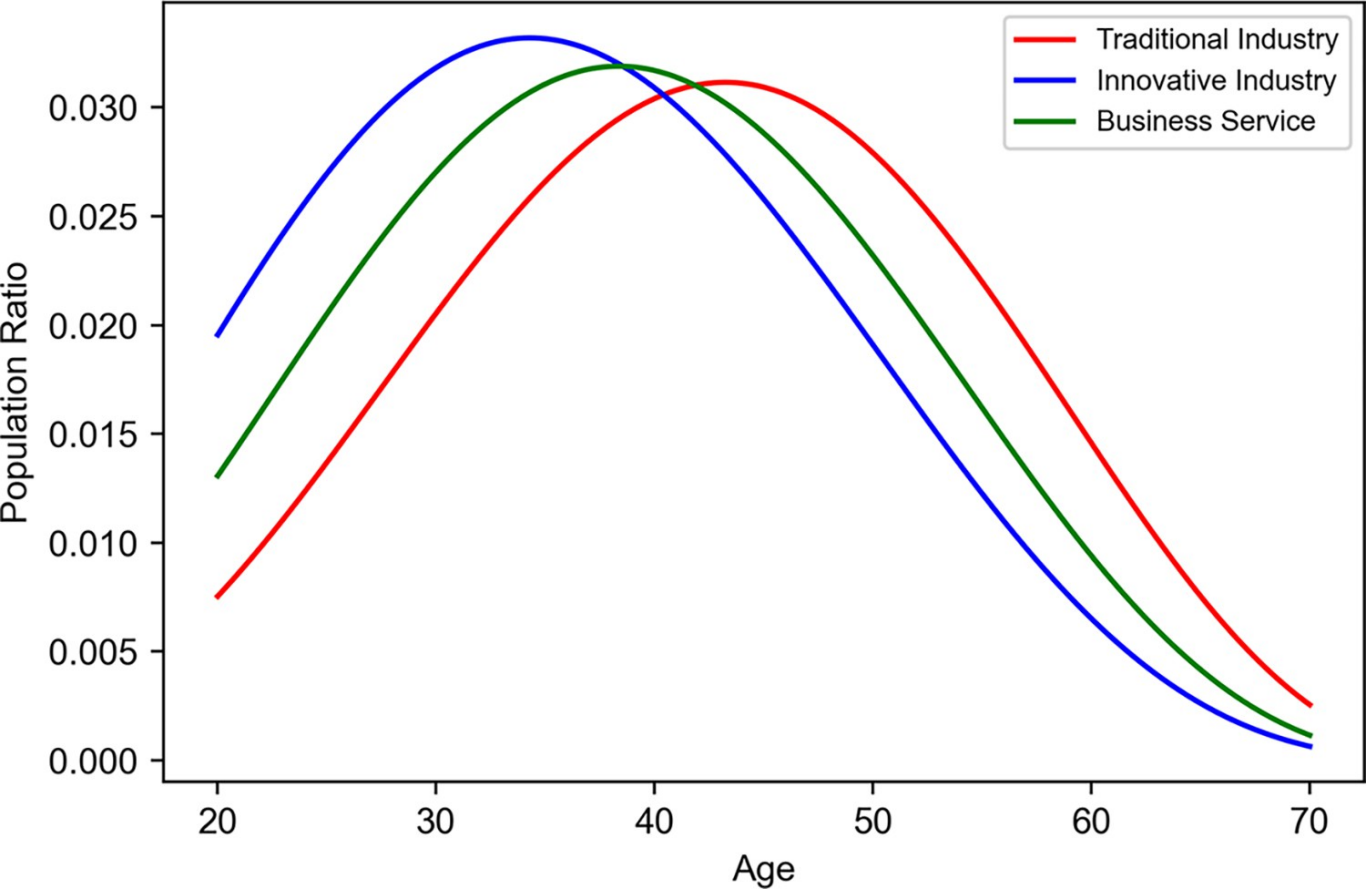
Initial population ratio for each age group.

Fig 7 presents the age distribution of the initial population across various economic sectors. It clearly delineates the concentration of different age groups within each sector, enabling a detailed analysis of the population structure and age-related economic participation within these sectors.

#### Cluster density index (CDI) model

The cluster density index (CDI) model serves as an indicator for measuring the spatial distribution density of entities. This index quantitatively represents the degree of concentration of entities within a specified distance and is, thus, utilized for analyzing spatial clustering tendencies. The formula of the model is as follows:

$$K(d)=\frac{\sum_{i}N_{i}\cdot no[S\in C(S_{i},d)]}{N}$$ (10)

*K(d)* signifies the cluster density and *N* the total number of workers. *C(S*_*i*, *d*_*)* denotes a circle at distance d from central point *S*_*i*_, and the density of the cluster is calculated based on the number of entities located within this circle [49]. In this study, a set distance of 10 served as a benchmark for measuring the extent of clustering, where a higher value of this index indicates a close concentration of entities, suggesting active spatial clustering. The CDI proved useful for understanding the general distribution in this study. For a more detailed analysis of clustering, the value of *K(d)* can be adjusted to apply a standardized index [1]. The CDI model enables the analysis of clustering patterns in economic activities, social interactions, and various geographical phenomena. For instance, the model can be employed to gauge the concentration of specific industries or residential areas within a city, providing crucial insights for decision-making processes in regional development strategies and for enhancing economic interconnectivity.

## Results

As previously mentioned, this study focuses on elucidating the interplay between agglomeration economics and congestion diseconomies by adjusting the congestion parameter *λ*. Simple and complex parameter experiments were conducted to explore the role of self-organizing regulatory mechanisms in urban environments. Our findings reveal that subtle variations in the value of *λ* trigger distinct patterns within the urban setting, notably the formation of clusters and the spread of sprawl. Specifically, a pronounced clustering phenomenon is observed when *λ* was below 0.11, while sprawl patterns emerge for *λ* values exceeding 0.12. Unique dynamic changes are observed near threshold values.

### Experiment 1: Oscillation and Wave in Simple Parameter

In “Experiment 1: Oscillation and Wave in Simple Parameter,” we conducted an in-depth analysis of the cluster, sprawl, and threshold area patterns represented by the *λ* values of 0.08, 0.15, and 0.11, respectively. This study is expected to make a substantive contribution to the effective establishment of urban planning and economic policies. Time series data for each *λ* value were collected from the perspectives of population, birth, development, and CDI. Analyzing various industrial sectors, this data provides crucial insights into the dynamic urban changes associated with different *λ* values.

#### Population

**Fig 8:**
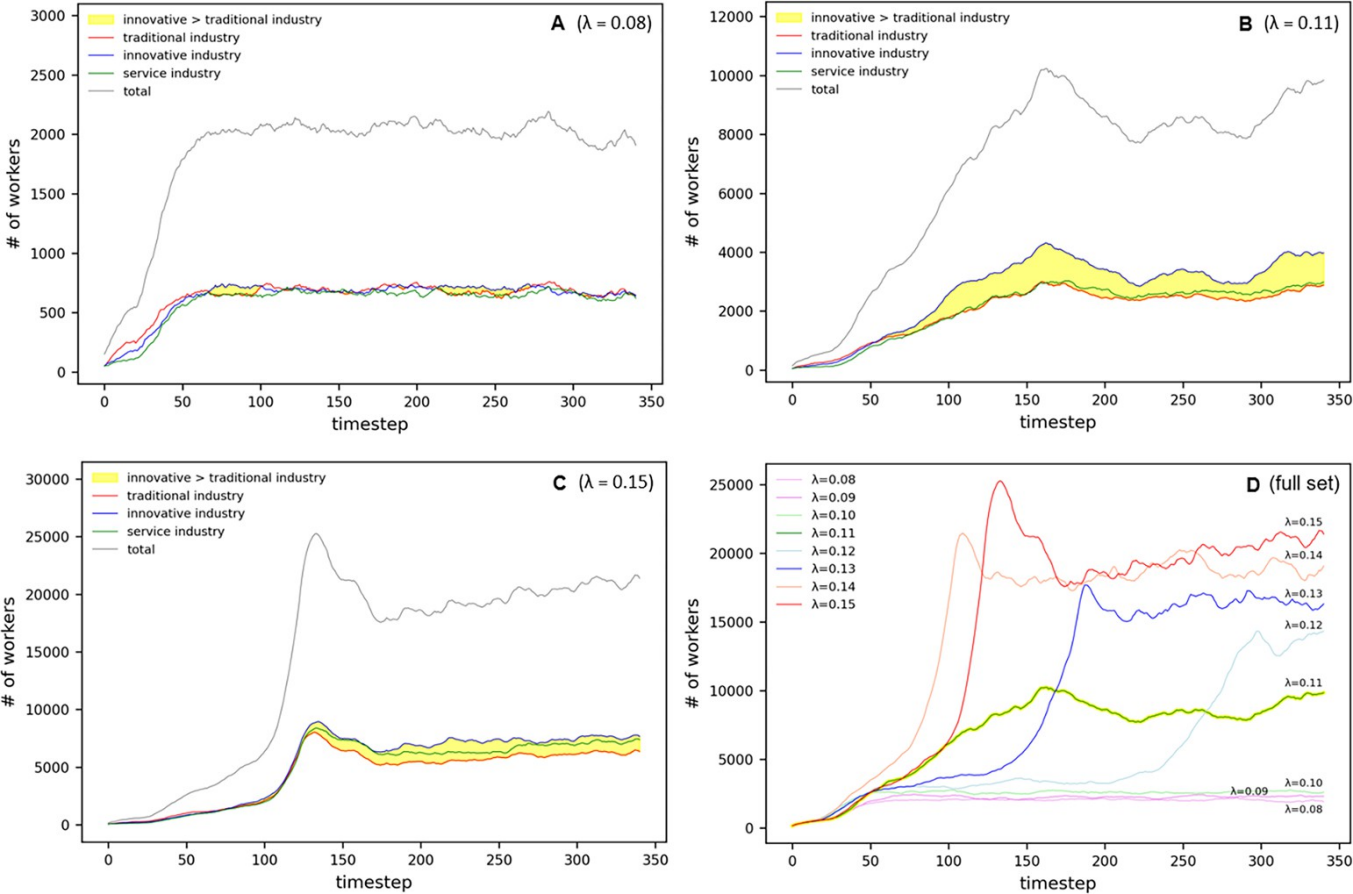
Population oscillation in Simple Parameter.

Our analysis demonstrates clear convergent trends in population aggregation patterns resulting from the adjustment of the congestion parameter *λ*. With a setting of *λ* = 0.08, the population stabilized at around 2,000 individuals (refer Fig 8A). Under these parameter conditions, each industrial sector achieves a balance with a similar population of approximately 600 individuals.

In the sprawl scenario, when applying *λ* = 0.15 (as illustrated in Fig 8C), the population initially surged, followed by a linear increase from *t* = 170 to *t* = 340, oscillating between 18,000 and 22,000 individuals. Sector-wise, traditional industries concentrated around 5,000 individuals, services around 5,500, and innovative industries around 6,000.

At the threshold setting of *λ* = 0.11 (see Fig 8B), the population peaked at timestep *t* = 160, after which the aggregation fluctuated between 10,000 and 8,000. Here, innovative industries oscillated between 4,000 and 3,000 individuals, while traditional industries and services remained at approximately 2,000 individuals. This trend reveals the interaction between the industrial sectors and population dynamics near the threshold.

The comparative analysis of population changes between the cluster and sprawl types (depicted in Fig 8D) showed that, while the cluster type exhibited a stable aggregation pattern of approximately 2,000 individuals, the sprawl type demonstrated a differentiated tendency, clustering between 14,000 and 21,000 individuals. This distinction underscores the boundary line between the two patterns at *λ* = 0.11, with the peaks observed at timesteps *t* = 290 and *t* = 340 at *λ* = 0.12, further clarifying this demarcation.

#### Birth

**Fig 9:**
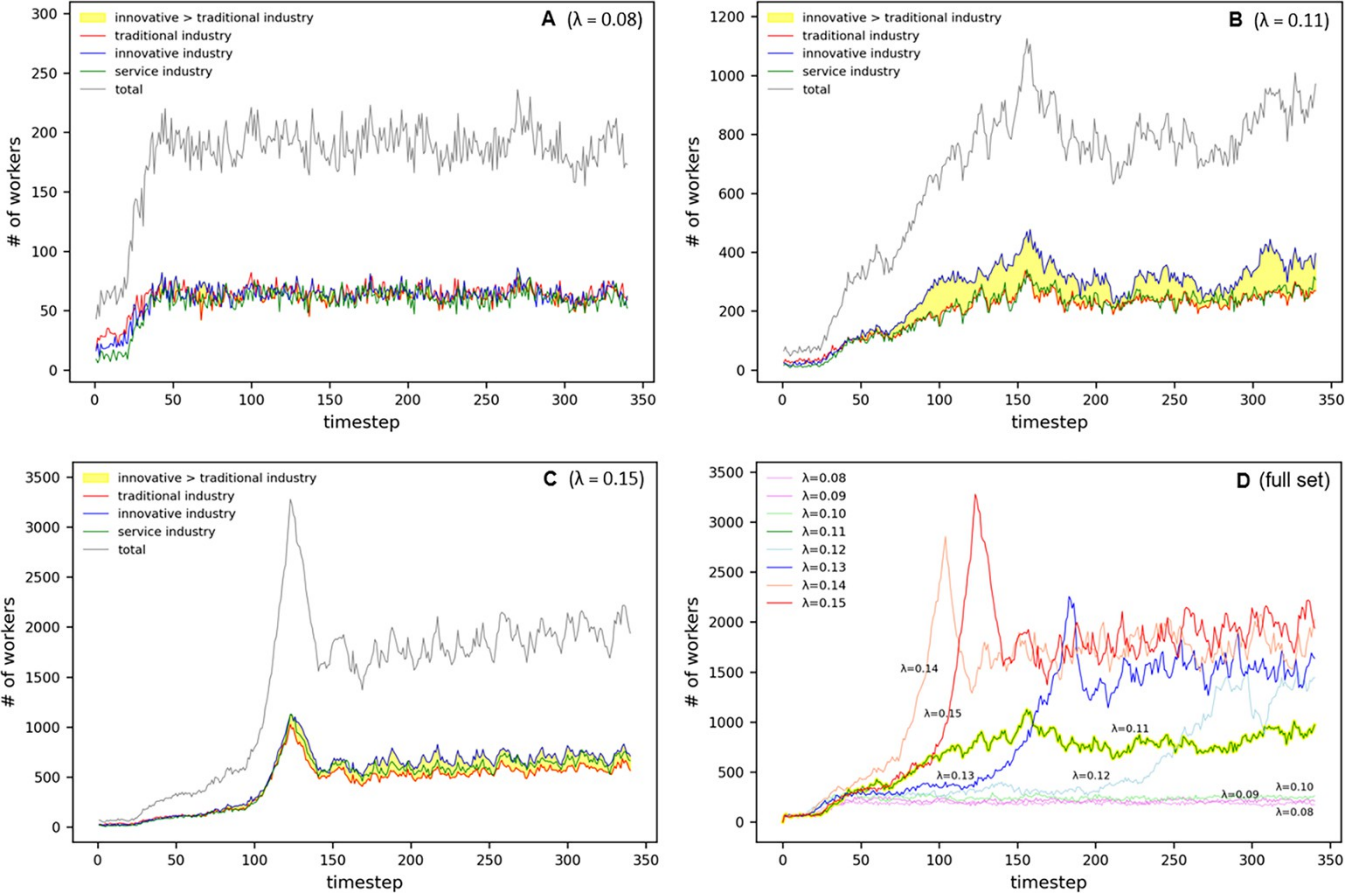
Birth oscillation in Simple Parameter.

For birth rates, a pattern similar to that of the overall population trend was observed (Fig 9). However, they are distinguished by pronounced oscillations and periodicity, which reflect the complex dynamics and fluctuating forces of urban growth.

#### Development

**Fig 10:**
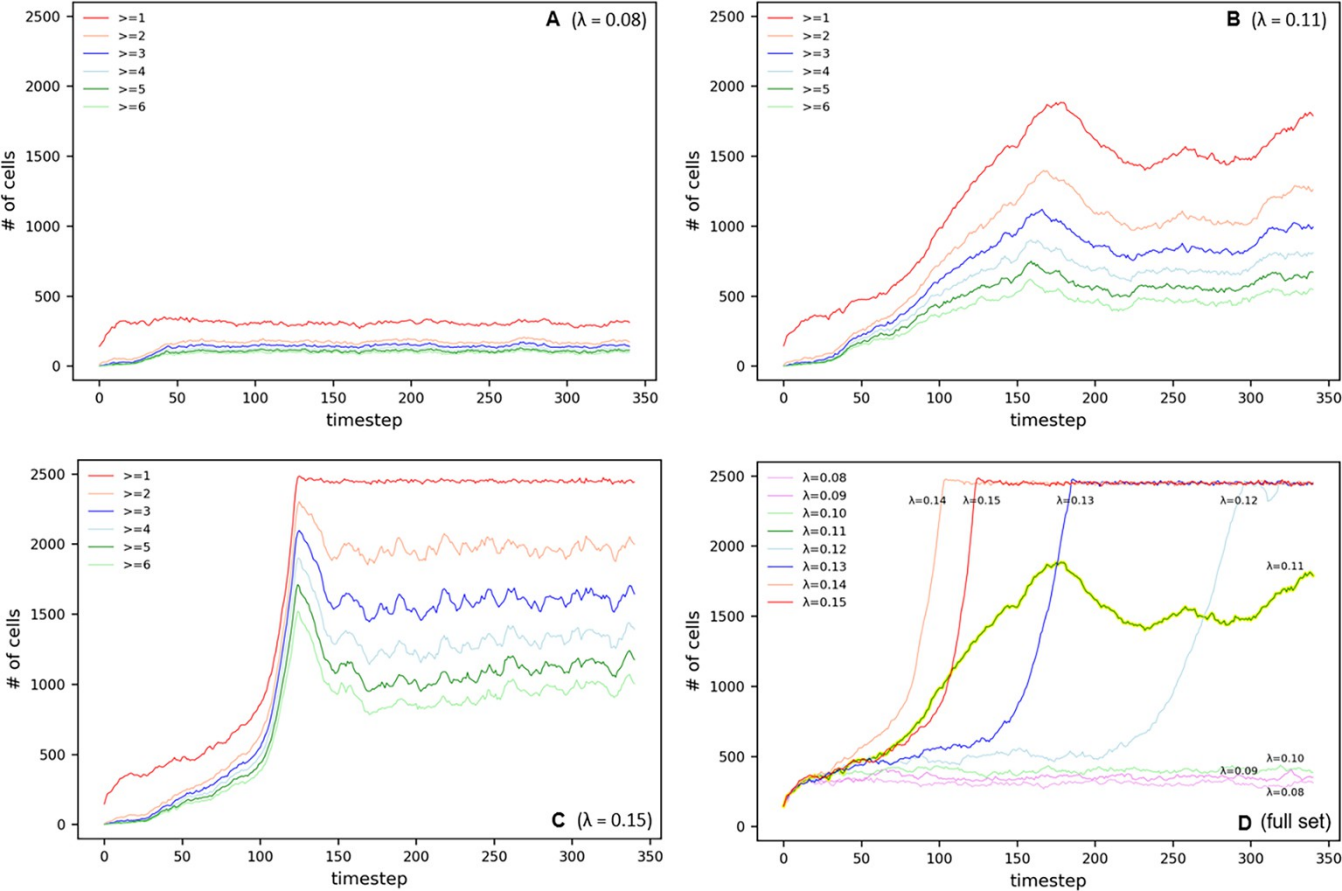
Development oscillation in Simple Parameter.

Distribution patterns of residential populations are pivotal variables in urban development. Our study identified the developmental states based on the number of working populations per cell through a time-series analysis, and the graphs in Fig 10 capture these patterns.

As exhibited in Fig 10A, the cluster-type development pattern allows us to discern the dynamic equilibrium in urban growth where, following an expedited population surge, the residential population stabilizes after a certain period. The stabilization observed at *t* = 40 signifies that the city has reached a state of equilibrium.

By contrast, the sprawl-type development pattern in Fig 10C shows a diffusive development trend where, after *t* = 120, the residential population becomes distributed across nearly all cells. This strongly indicates a trend in which the city, after initial agglomeration, undergoes continuous expansion, thereby utilizing a wider expanse of urban space.

The analysis in Fig 10B identifies a critical threshold at *λ* = 0.11, where urban development represents a complex equilibrium between clustering and sprawl. This is a significant indicator for forecasting the nuanced shifts that may occur during urban spatial development.

Through a comprehensive analysis of the parameters, it is apparent that the cluster type tends toward stabilization within a confined developmental scope, whereas the sprawl type distinctly reveals a trend of exploiting the overall urban space through ongoing expansion. This suggests the necessity of considering the balance between concentration during the initial stages of growth and subsequent dispersion when formulating urban planning and spatial strategies.

#### CDI

**Fig 11:**
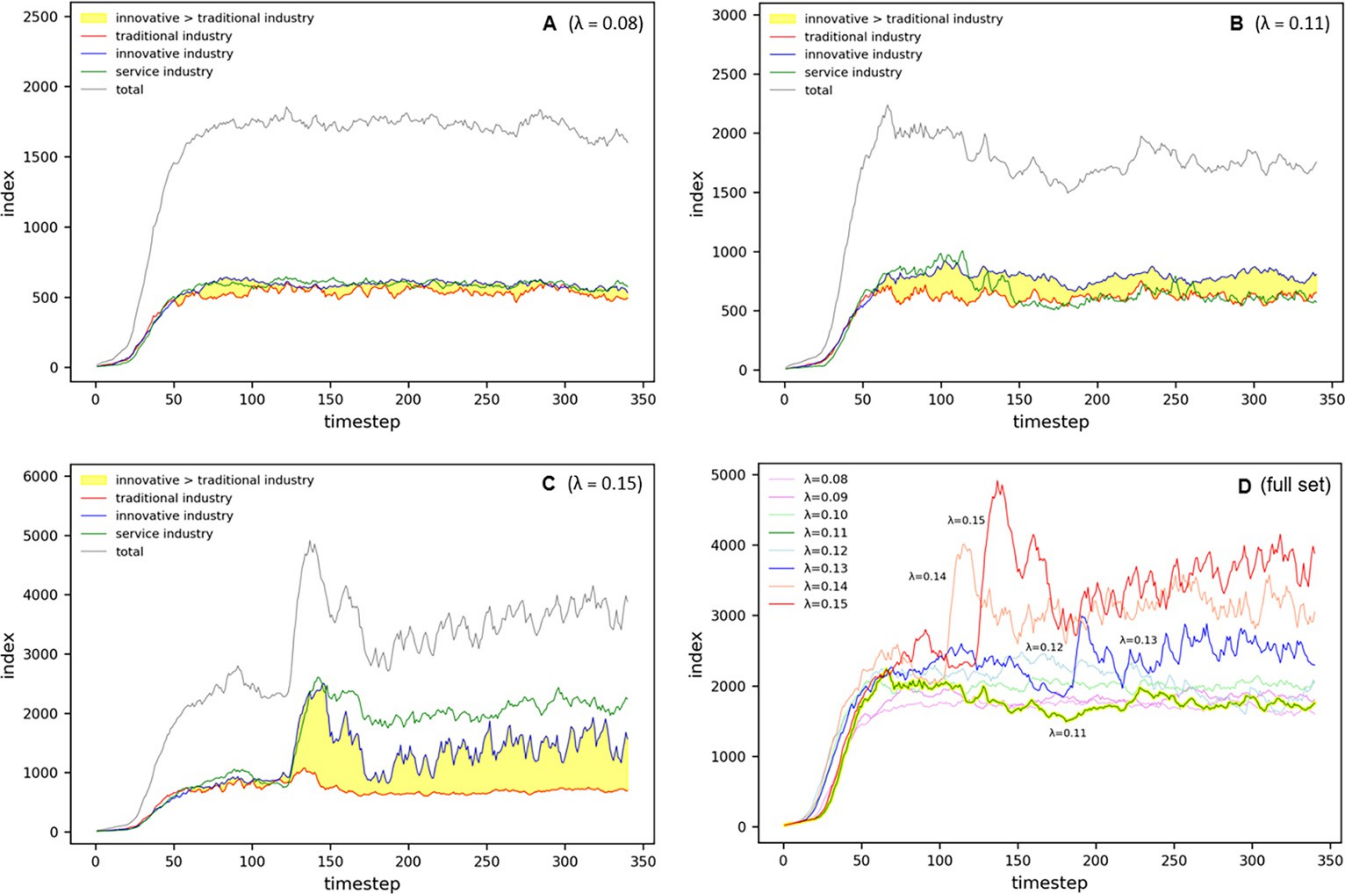
CDI oscillation in Simple Parameter.

The CDI, an essential tool in urban planning and development research, quantitatively measures the spatial agglomeration and development of cities. Through a time-series analysis, as indicated in Fig 11, this study illustrates the dynamism of the urban CDI as influenced by fluctuations in the *λ* value.

The cluster type of urban development tends to maintain a relatively stable agglomeration within specific regions, as confirmed by the analysis in Fig 11A. Conversely, the sprawl type of urban development exhibits an oscillatory trend toward stabilization after an initial sharp rise in the CDI within the service sector, as observed in Fig 11C.

In the critical region CDI analysis, urban space at *λ* = 0.11 appears to be poised at a balance point between dynamic change and stable development, as shown in Fig 11B. This phenomenon contrasts with the volatility exhibited by the other urban development indicators.

The comprehensive parameter analysis in Fig 11D shows an unexpectedly low CDI at *λ* = 0.11, particularly experiencing a significant decline at *t* = 180. This suggests that urban cluster density decreases around this critical threshold, marking an important juncture that reflects shifts in urban structure and development patterns.

**Fig 12:**
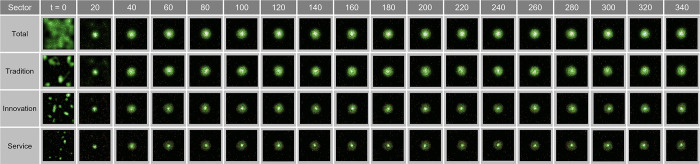
Wave in congestion λ = 0.08.

Based on Fig 12, we have conducted an in-depth examination of the spatial oscillations manifesting in urban spaces at the state of *λ* = 0.08. This provides baseline data for quantitatively discerning central agglomeration phenomena and their determinants in urban modeling studies. The findings in Fig 12 validate the propensity of the traditional, innovative, and service sectors to pursue concentration toward the city center up to *t* = 340. Specifically, the traditional industry exhibited diffusion toward the periphery of the clusters, while the innovative industry showed a higher degree of central concentration, and the service sector revealed a very pronounced pattern of central agglomeration.

According to Fig 11A, in the state of *λ* = 0.08, the CDI for innovative and service industries is relatively higher than that for the traditional industry, indicating that these sectors are enhancing the city‘s innovative capacity and service orientation through agglomeration in the urban core.

**Fig 13:**
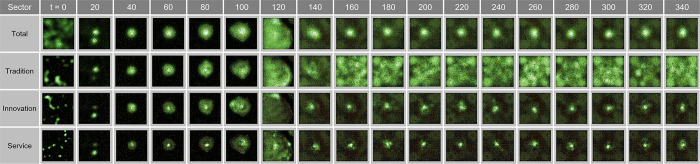
Wave in congestion λ = 0.15.

This study analyzes the specific oscillatory dynamics of urban spaces under the condition of *λ* = 0.15, shedding light on the intricate aspects of urban agglomeration. As observed in Fig 13, the traditional, innovative, and service sectors exhibit a concentrated development pattern around their distinct clusters.

At *t* = 120, the traditional industry in the north begins to disperse from the initial agglomeration, contributing to cluster formation, whereas the innovative industry and service sector in the south maintain a high-concentration development pattern in the cluster centers (*t* = 100). From this point on, the sub-centers within the clusters show a trend of progressive integration and expansion.

Further analysis after *t* = 100 reveals that, while the traditional industry shows active dispersion in the southeast of the cluster, the innovative industry experiences concentrated growth in two sub-centers within the cluster, and the service sector displays a pattern of emptying the surrounding donut-shaped areas while concentrating in the core.

In the long term, from *t* = 120 to *t* = 340, traditional industries tend to disperse throughout the region, evolving into a conurbation-like form, whereas the innovative industry and service sector display simultaneous central agglomeration and peripheral dispersion, with certain areas around the core uniquely excluded from the spatial concentration.

**Fig 14:**
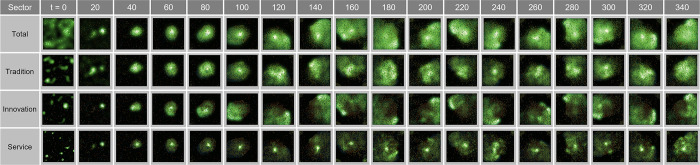
Wave in congestion λ = 0.11.

Fig 14 illustrates the oscillatory changes in urban spaces under the condition of *λ* = 0.11. Starting from the city’s initial random distribution state, the two distinct agglomeration centers observed at *t* = 20 distinctly reveal the spatial preferences among the traditional, innovative, and service industries. This contrasts the pattern of traditional industry clustering in the west, while the eastern center shows a more concentrated clustering of innovative and service industries. Over time, these centers gradually integrate, particularly with innovative industry positioned at the outskirts of the cluster, and the service sector exhibits a pronounced agglomeration pattern in the cluster core while leaving the surrounding donut-shaped areas empty (*t* = 100).

The detailed analysis at *t* = 160 indicates a complex structure, in which the service and traditional industries are concentrated at the geographical center of the cluster, while the innovative industry is concentrated in the peripheral sub-centers. With some service industries located in the peripheral centers, this allows for a clearer understanding of the industry-specific distribution within urban spaces and the dynamic changes in agglomeration. This demonstrates that the spatial structures of cities can be influenced by the gravitational forces of industries over time.

#### Global Moran’s I analysis

This study quantitatively assessed the spatial autocorrelation of urban economic activity using the Global Moran’s I and Local Moran’s I indices. The experiment was conducted under “Simple Oscillation” and “Congestion” conditions to analyze spatial patterns for three parameter settings: λ = 0.08, 0.11, and 0.15.

**Fig 15:**
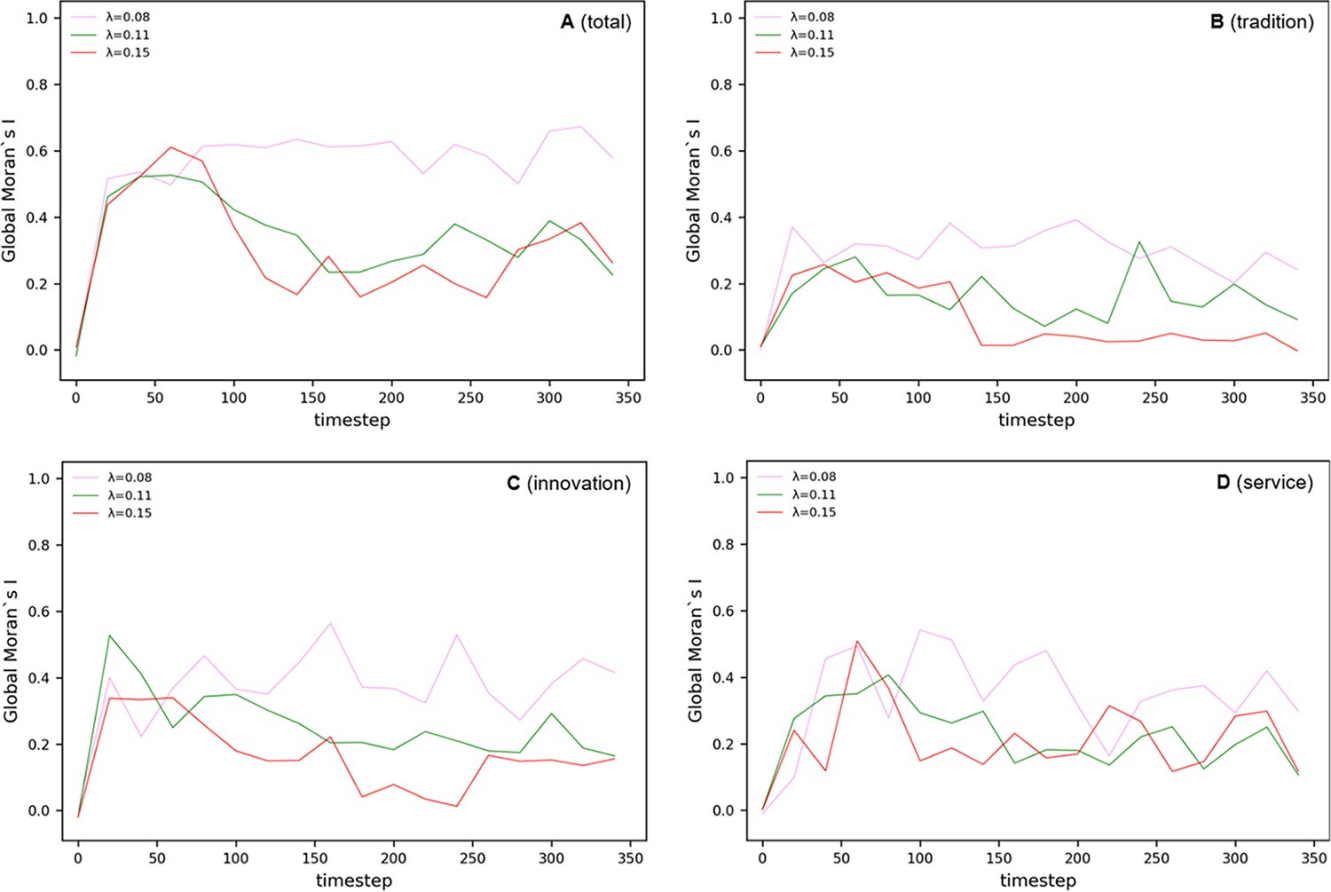
Global Moran’s I in Simple Parameter.

Fig 15 shows the change in Global Moran’s I index over time under the Simple Oscillation experiment. Starting from λ = 0.08, a trend can be clearly observed where the spatial clustering of overall economic activity decreases with increasing lambda values. This downward trend is most evident in traditional industries, even as the service sector maintained relatively high stability. These results have important implications for understanding the impact of economic policies on spatial distribution under the congestion condition.

#### Local Moran’s I analysis

**Fig 16:**
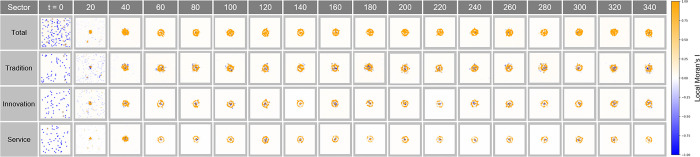
Local Moran’s I in congestion λ = 0.08.

Fig 16 shows the results of analyzing the Local Moran’s I index under the congestion condition at λ = 0.08. The distinctive donut-shaped pattern on this map indicates that congestion in the city center is driving economic activity to the surrounding areas. In particular, the high index value in the outskirts, as opposed to the low index value in the center, suggests that clustering is dispersing to the outskirts to avoid congestion in the center. This has important implications for reassessing the economic interaction and connectivity between the city’s core and its outskirts.

**Fig 17:**
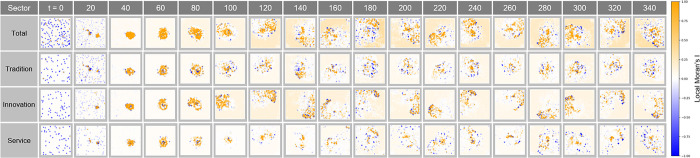
Local Moran’s I in congestion λ = 0.11.

Fig 17 reflects the congestion state at λ = 0.11. Economic clustering becomes more evident in the outskirts, where the Local Moran’s I index is higher, suggesting that the city center is losing its function as a core area of economic activity. This distribution shows the potential for urban planning to concentrate economic activity in areas away from congestion, suggesting that economic activity moving from the center may form new clusters.

**Fig 18:**
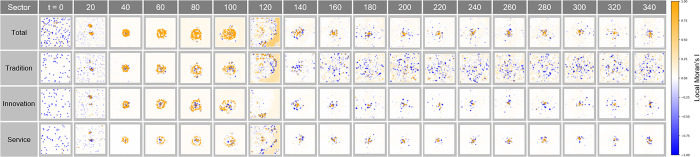
Local Moran’s I in congestion λ = 0.15.

Fig 18 visualizes the Local Moran’s I index under the congestion condition observed at λ = 0.15. Higher values of this index indicate a clear shift of the center of economic activity to the outskirts of the city. This is a strong indicator that in urban economic patterns, the core is transforming into an area excluded from economic activity, and the outskirts are emerging as new economic centers. This information serves as an important reference for understanding urban economic congestion and the resulting clustering phenomenon, managing congestion, and making strategic interventions to revitalize the city’s economy.

Figs 16–18 provide a detailed spatial representation of how the Local Moran’s I index changes with the value of λ under the congestion condition. These indices capture a donut-shaped pattern in which economic activity in a given region is more concentrated in the outskirts than in the center. For λ = 0.08, 0.11, and 0.15, the spatial concentration of economic activity tended to increase with distance from the center, suggesting that there is a decentralized nature of economic activity that avoids congestion in the center. These analyses provide an important basis for urban planners to develop strategies that can simultaneously pursue congestion management and economic revitalization.

#### Getis-Ord G* analysis

This study utilized the Getis-Ord G* statistic to evaluate the concentration of spatial clustering under various congestion conditions. This statistic is used to identify the location of high- and low-density clusters in a specific area. Experiments conducted for values of λ = 0.08, λ = 0.11, and λ = 0.15 provide a visual representation of how economic activity within a city is readjusted in response to changes in congestion levels.

**Fig 19:**
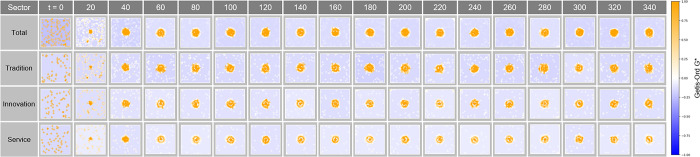
Getis-Ord G* in congestion λ = 0.08.

In Fig 19, the results reflecting the congestion state of λ = 0.08 showed that high-density clusters tended to be distributed relatively evenly throughout the city. Overall, clustering is slightly more pronounced in the outskirts than in the center, which may indicate the early stages of a shift of economic activity from the urban core to the periphery due to congestion. This reveals that traditional industries form strong clusters in central regions, while innovation and service industries expand into outlying regions.

**Fig 20:**
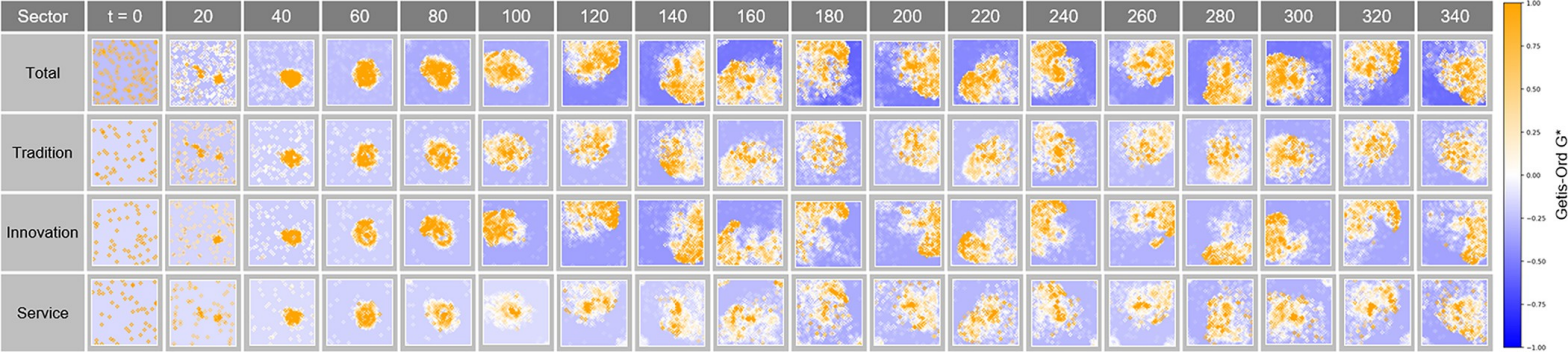
Getis-Ord G* in congestion λ = 0.11.

Fig 20 suggests high density clusters of economic activity observed under the congestion condition at λ = 0.11. This map shows that economic activity is becoming more concentrated in outlying areas, corresponding to higher values of λ. In particular, clusters of innovation and service industries appear more clearly in the outskirts compared to the core, showing that central congestion affects the spatial distribution of these industries.

**Fig 21:**
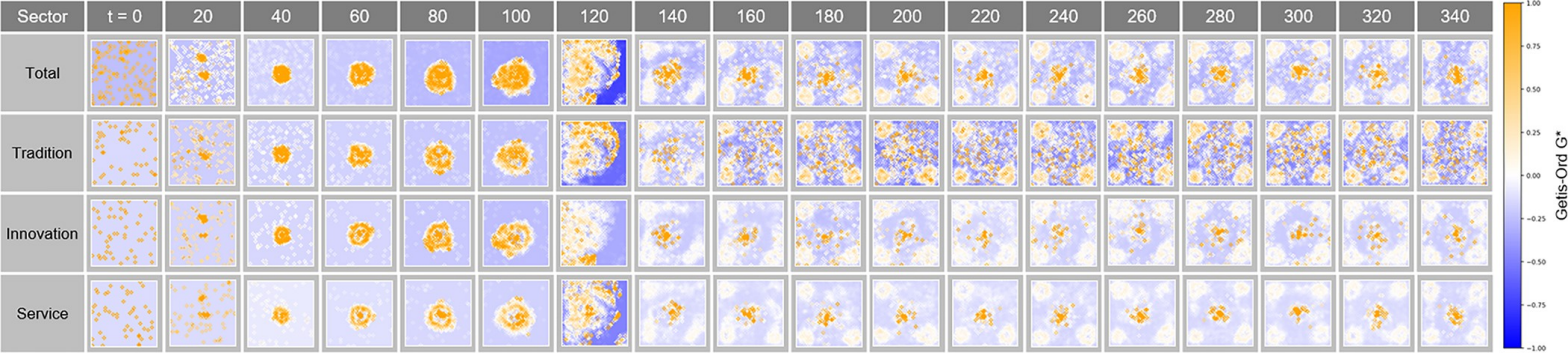
Getis-Ord G* in congestion λ = 0.15.

In Fig 21, the pattern revealed by the Getis-Ord G* statistics under the congestion condition of λ = 0.15 is more clearly revealed. Overall, the size and intensity of clusters increase, indicating that clustering of economic activity is forming clear regional patterns as congestion becomes more sophisticated. While traditional industries still maintain strong central clustering, innovation and service industries show enhanced clustering in the outskirts of the city, strongly suggesting that these industries are moving to the outskirts to avoid central congestion.

This Getis-Ord G* analysis, together with the Global Moran’s I and Local Moran’s I results, contributes to a comprehensive understanding of the spatial distribution and clustering patterns of economic activity within cities. The tendency for economic activity to shift from the center to the periphery as congestion increases is an important trend on which urban planners and policymakers should focus. This information provides valuable insight into developing strategies for sustainable urban development and economic revitalization.

**Fig 22:**
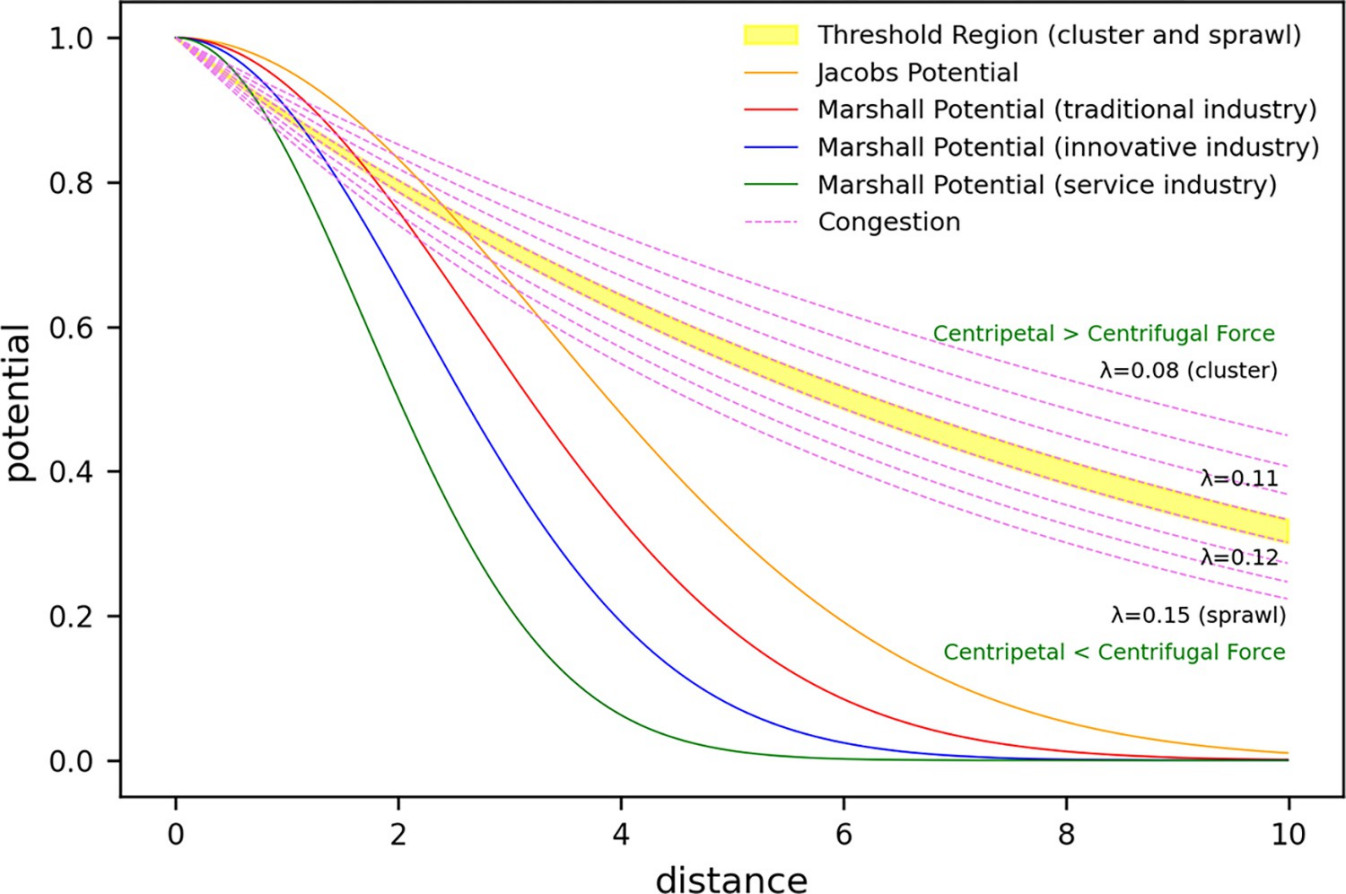
Potential and congestion plot.

Fig 22 models the effects of centripetal and centrifugal forces within urban structures on potential, offering a quantitative interpretation of the core mechanisms in urban planning that orchestrate the distribution of populations and economic activities.

According to these graphs, a lower *λ* (e.g., 0.08) intensifies the centripetal force, indicating a higher developmental potential in the urban core, which gradually diminishes toward the periphery. This suggests that the city center acts as a gravitational field and powerfully attracts economic agents and activities. Conversely, at a higher *λ* (e.g., 0.15), the centrifugal force becomes more pronounced, leading to a noticeable diffusion of activities toward the outskirts. This emphasizes the urban sprawl phenomenon, with congestion and rising costs in the center acting as catalysts for the development of the peripheral areas. At intermediate *λ* values (e.g., 0.11), the interplay of centripetal and centrifugal forces creates more complex patterns, where the city’s potential finds a balanced adjustment between the center and the periphery.

Such an analysis is paramount to comprehend the correlation between a city’s developmental potential and its level of congestion. By accurately assessing the spatial distribution of the potential and influence of the congestion parameters, we can formulate balanced urban development strategies and promote sustainable growth. This is particularly crucial at critical thresholds, where structural changes in the city are anticipated, providing essential data for predicting shifts in cluster density and development patterns.

This study has derived a detailed understanding of the interrelationship between urban agglomeration economies and congestion parameter *λ*, held constant in our simple oscillation and wave experiment. Notably, a critical threshold of *λ* = 0.11 has been identified as the pivotal region that forms the optimal urban state in the dynamism and interplay of urban agglomeration economies. Near this critical value, the manner in which urban spatial economies generate oscillations and waves, thereby actualizing the potential for urban development, is provided with depth and insight.

We thus recognize that cities are in a continuous state of change and emphasize that strategic interventions in economic, spatial, and demographic dimensions are essential to facilitate such transformations. We argue the importance of continuously shaping and reshaping urban congestion patterns through initiatives such as attracting new industries, investing in transportation infrastructure, and drawing on knowledge workers.

Through an experimental approach, our study dynamically adjusts fixed congestion parameter *λ* to reinterpret the interaction with agglomeration economies in a realistic context. Following our proposed methodology, complex oscillation and wave experiments are conducted to visualize the potential for urban development and changes associated with congestion parameter *λ*, meticulously analyzing the interplay of centripetal and centrifugal forces. This analysis deepens our understanding of urban planning and economic development models and provides critical insights for effective urban management and coordination strategies.

### Experiment 2: Oscillation and Wave in Complex Parameter

The second experiment conducted in this study, termed “Oscillation and Wave in Complex Parameter,” investigated the multidimensional impacts of variations in the urban congestion parameter *λ*. Designed over a temporal sequence of 740 timesteps with adjustments in *λ* every 50 timesteps, the experiment methodically decreased and then increased *λ* values from 0.15. This modeled a scenario in which congestion incrementally increased from *t* = 0 to *t* = 400, and the opposite decreasing scenario from *t* = 400 to *t* = 740, thus simulating the intricate dynamics within a variable urban context.

**Fig 23:**
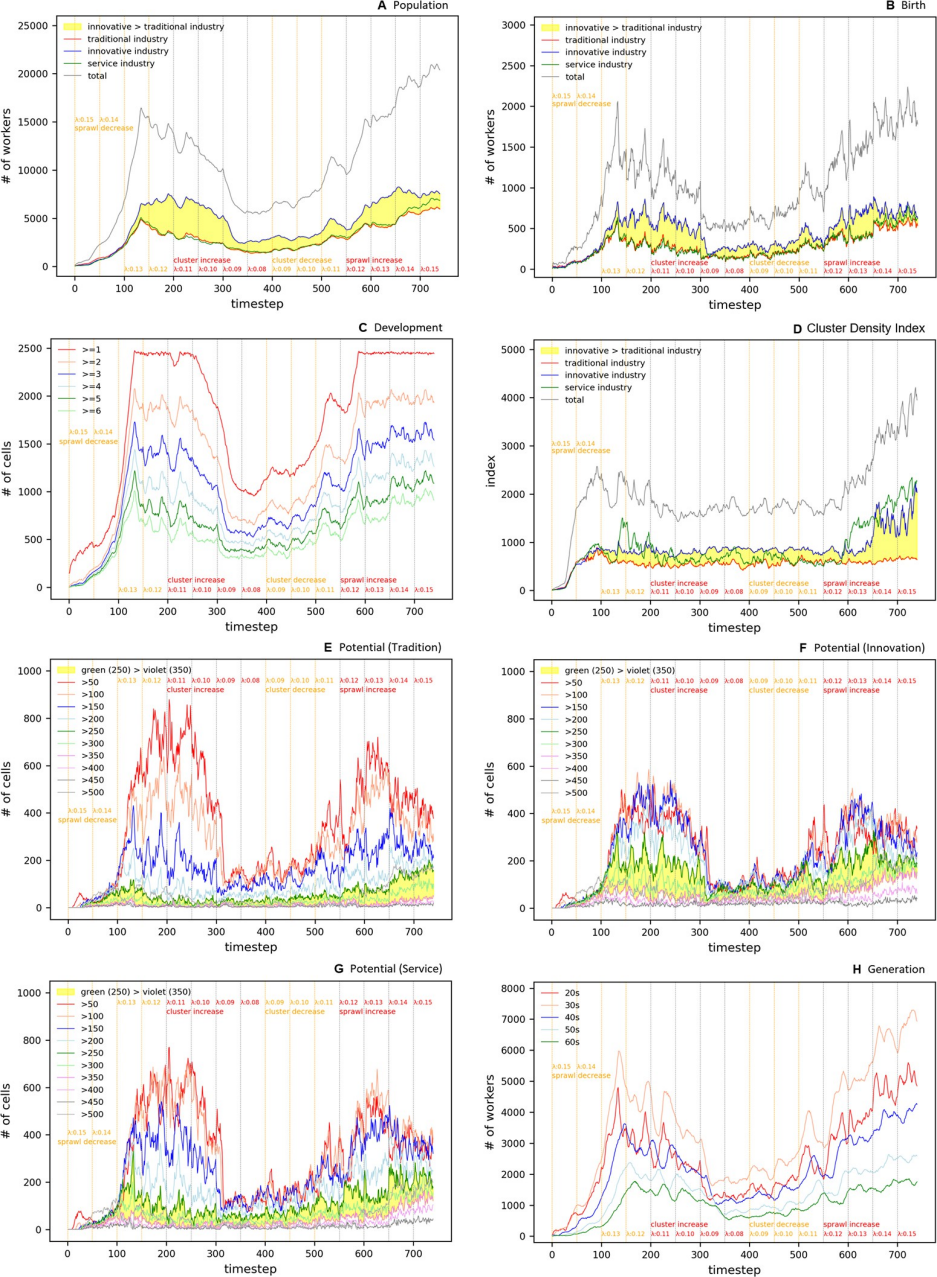
Oscillation in Complex Parameter.

Fig 23 represents the temporal variability of population, birth rates, development, CDI, and the potential of specific industrial sectors under these experimental conditions. Each subgraph intricately details the effects of the dynamic changes in the congestion parameter on these indicators.

In the complex oscillation experiment analysis, the overall population change exhibited three distinct phases. In the first phase, the total population gradually increases, reaching a peak at *t* = 140. This increase is primarily driven by the potential of agglomeration economies, with a steady rise until *t* = 100, before a sharp escalation (Fig 23E–23G). This surge reflects the entities concentrated in the core, expanding the scope of the cluster, transitioning into sprawl phenomena at *t* = 120 with the formation of two centers, and converging into a single center at *t* = 140, revealing complex dynamic patterns.

The traditional industry exhibits a peak in population at *t* = 120, followed by a downward trend, indicating a limitation in birth rates due to the low potential across most cells (Fig 23E). During complex oscillation and wave, as the population approaches its peak, three centers form within the cluster core, increasing to four at *t* = 120 with sprawl emerging in the northern region and intensifying across the entire cluster at the peak. Notably, small-scale clusters of low-density form in the northeast and northwest regions.

The innovative industry tends to maintain its scale even after reaching its peak, suggesting its ability to sustain high birth rates owing to its robust potential (Fig 23A, 23B, 23F). Throughout the complex oscillation and wave, the centers of the innovative industry and entities remain consistent, indicating a preference for geographical accessibility despite significant congestion.

**Fig 24:**
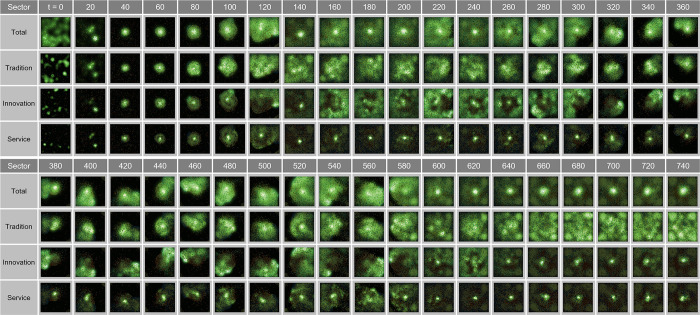
Wave in Complex Parameter.

The service sector displays a pattern similar to that of the traditional industry in terms of both population and birth rates, showing a decline after reaching the peak. However, its spatial distribution initially sprawls into low-density clusters around the core until *t* = 120, and then dissipates at *t* = 140 (Fig 24).

Such complex dynamics have significant implications for urban planning and policy decision-making, providing essential insights for strategic planning necessary for sustainable urban growth. Our findings detail the intricate impacts of dynamic changes in the congestion parameter on various aspects of the city, offering critical perspectives not only on macro-urban changes but also on micro-level shifts across industries and the consequent reconfiguration of urban structures.

In the second interval of population change, spanning *t* = 160–360, the observed complex oscillatory phenomena were analyzed in detail. The total population displays two instances of oscillation at approximately *t* = 180 and *t* = 210; however, the underlying trend is one of decline. These oscillations, which occur upon reaching the threshold area of lambda values at 0.12 and 0.11, are interpreted as a natural response of seeking homeostasis within the ecosystem (refer to Fig 24).

In the analysis of spatial distribution patterns, an excessive concentration of entities is observed in the center of the world at *t* = 140, forming a high-density core within the city center. Subsequently, a consistent urban sprawl phenomenon persists until *t* = 300, leading to the division of the central urban area into two independent clusters by *t* = 320. The continued decrease in population triggers the reintegration of clusters from *t* = 340, fostering a reduction in cluster size and promoting an uneven distribution pattern (Fig 24).

The population in the traditional industries and service sectors exhibit oscillatory behaviors between *t* = 180 and *t* = 210, declining at similar rates, yet their spatial distributions reveal contrasting patterns (Fig 24). While the traditional industry maintains an undefined sprawl, the service industry is predominantly concentrated in the center of the world.

Notably, the distribution of the innovative industry from *t* = 160 to *t* = 280 exhibits a wave-like form, predominantly located in donut-shaped areas encircling the city center, akin to ripples created by a droplet in a pond (Fig 24). This suggests that the innovative industry, by situating itself both within and on the periphery of the city, indicates the dynamic nature of the urban structure and population distribution.

During the third interval from *t* = 380 to *t* = 740, marked by a resurgence of congestion, the spatial patterns of the entire sector exhibit two distinct behaviors. Between *t* = 380 and *t* = 560, some centers emerge and vanish rapidly, a phenomenon attributed to entities swiftly relocating to find suitable locations in a state of high congestion. Notably, a decrease in the number of entities, coupled with a decline in the persistence of cell development, lead to a quick transition to a vacant state. However, from *t* = 580 to *t* = 740, an intensification of clustering in the urban core is observed, interpreted as a resurgence in population due to a significant increase in entities boosting the core’s potential and, consequently, through the feedback system of birth rates.

For the traditional industry, a gradual increase in population is observed, with minimal oscillations even in the threshold region around *t* = 500–550. Starting from the descent in congestion at *t* = 580, a polycentric pattern begins to emerge and, after *t* = 660, dispersal toward the outskirts leading to a vacating urban core is observed. Throughout this process, entities tend to cluster loosely rather than form definitive agglomerations.

In the innovative industry, the population increases substantially more than in the traditional or service sectors. This correlates with the rise in cells with high potential, as depicted in Fig 23F, suggesting strategic adjacency, where a significant number of entities avoid congestion. Fig 24 reveals a pattern of initial clustering in the outskirts or periphery until *t* = 560, the formation of waves within *t* = 580–620, and re-clustering in the urban core after *t* = 640. As congestion diminishes and the traditional industry spreads outwards, the innovative industry tends to regroup toward the center.

The service sector demonstrates a sprawl effect toward the periphery during the high-congestion period of *t* = 380–580, followed by reconcentration in the urban core. This transition is verified in Fig 24.

Figs 23 and 24 illustrate the complex interplay between sectoral spatial distribution and population dynamics within the city, responding to changes in congestion levels. These insights are pivotal in establishing urban planning and economic development policies.

#### Global Moran’s I analysis in Complex Parameter

**Fig 25:**
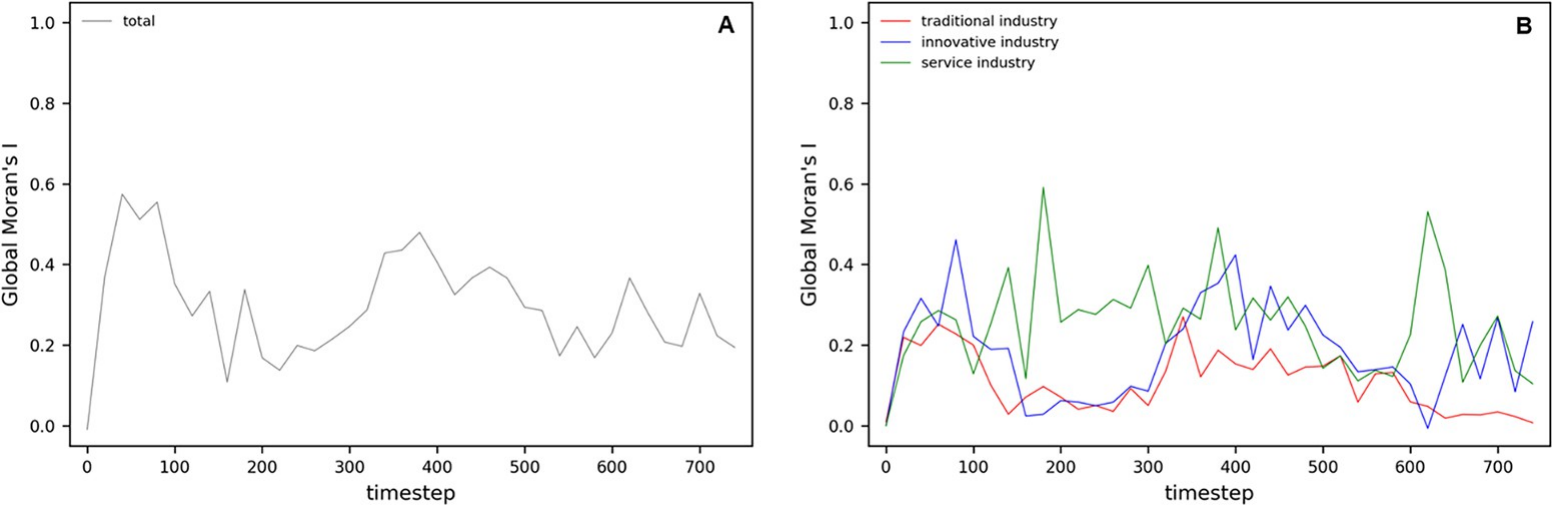
Global Moran’s I in Complex Parameter.

The Global Moran’s I index in Fig 25 tracks the spatial autocorrelation of overall economic activity changes over time t. Fig 25A shows the volatility of the overall index over time, which can reflect the impact of various economic events and external shocks on economic patterns. Fig 25B shows how the Global Moran’s I index for traditional, innovative, and service industries responds differently with time. This demonstrates how each industry adapts differently to market changes and policy impacts over time. Furthermore, the strength of spatial autocorrelation experienced by each industry at any given time is evident.

#### Local Moran’s I analysis in Complex Parameter

**Fig 26:**
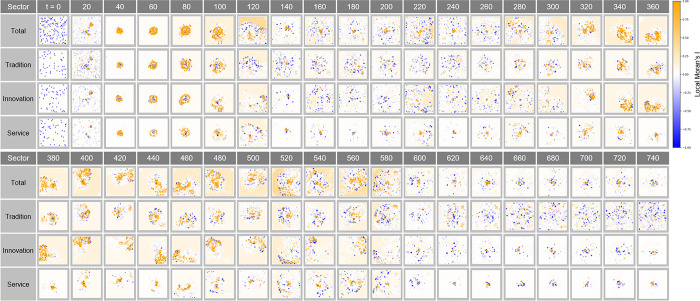
Local Moran’s I in Complex Parameter.

Fig 26 shows the local clustering pattern of economic activity over time t. Here, the distribution of Local Moran’s I index at each time step provides a visual representation of how the city’s spatial economic structure is changing. A series of clusters tends to expand or contract over time in synchronization with economic cycles, demonstrating the impact of factors such as policy changes, industrial developments, or technological innovations on localized concentrations of economic activity.

#### Getis-Ord G* analysis in Complex Parameter

**Fig 27:**
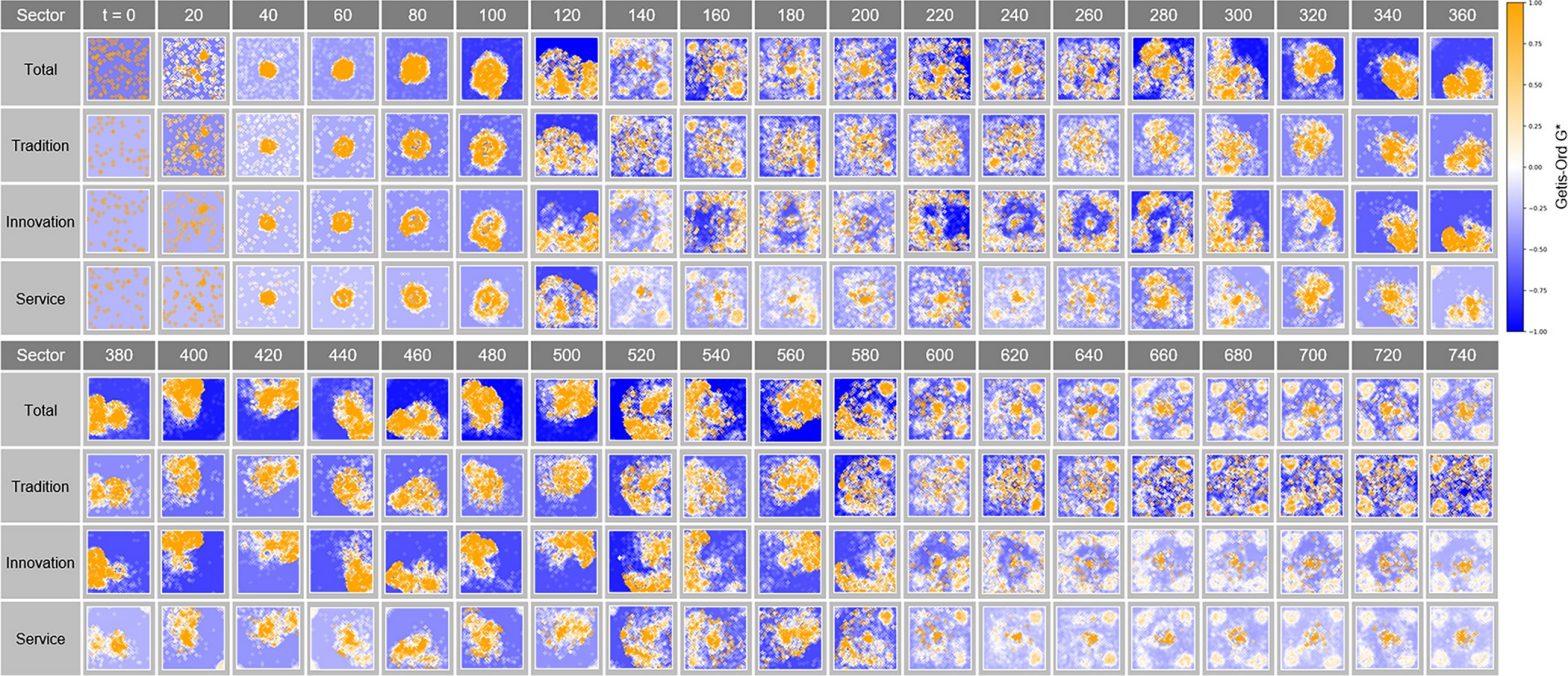
Getis-Ord G* in Complex Parameter.

The Getis-Ord G* analysis in Fig 27 tracks the evolution of the concentration and shape of spatial clusters over time t. The changes in hotspots and cold spots at each time step show how market dynamics and clustering patterns adjust over time. In particular, this analysis indicates how economic clusters respond to economic incentives or external shocks, which provides important information about how economic connectivity within urban space develops or is rearranged over time.

Finally, by identifying how a city’s cluster density and development patterns shift near the threshold values, this study underscores the need to consider urban dynamics in policy decisions for sustainable urban development. It also presents empirical and strategic directions for addressing the complex issues faced by cities, reflecting dynamic changes in competition and interactions between industries within the urban structure.

### Sensitivity analysis

Establishing and implementing policies for the urban economy is a complex task; to deepen our understanding of this task, carefully analyzing the interactions and influence of each policy element is necessary. The sensitivity analysis presented in this paper measures the impact of changes in the weights (ρ) of the Jacobs potential (JP) and Marshall potential (MPtras, MPinnos, MPservice) on employment, based on an extensive dataset obtained through urban economic simulations. By identifying the relative importance of each potential and the sensitivity of weight adjustments in determining changes in urban employment, this analysis provides important insights for formulating policies to maximize economic attractiveness and industrial agglomeration effects.

The basic functional form used in this subsection is expressed as Eq (11). Here, UP refers to the potential of urban economic activity, each β represents the default weight of that potential, and ρ represents the variable weight adjusted for sensitivity analysis. This function quantitatively models the impact of changes in potential weights on overall employment and employment in each economic sector under different scenarios.

Additionally, Eq (11) applied in this sensitivity analysis includes the congestion parameter λ, which plays an important role in evaluating UP. In this study, 0.11 was chosen as the value of λ, which was determined based on historical data from economic simulation models and realistic economic conditions. Considering other values such as 0.08 and 0.15, 0.11 was judged to most accurately reflect the congestion costs associated with the city’s infrastructure capacity. This value is expected to provide an appropriate standard for realistic urban planning and policy development by avoiding overly conservative estimates while adequately representing the negative impacts of urban space constraints and resulting congestion on economic activities.


$$UP=\rho_{1}(\beta_{1}IP)+\rho_{2}(\beta_{2}MP_{\mathrm{tras}})+\rho_{3}(\beta_{3}MP_{\mathrm{innos}})+\rho_{4}(\beta_{4}MP_{\mathrm{service}})-\beta_{5}\mathrm{CON}$$ (11)

The potential of a city’s economy affects employment patterns through complex interactions. Sophisticated sensitivity analyses are essential to understand this potential. In this subsection, we seek to evaluate the impact of adjusting the weights of the JP and MPtras, MPinnos, MPservice on employment changes. To this end, Eq (11) was used to quantify the impact of changes in the weights of various potential indicators on overall employment and employment by economic sector.

The number of employed people derived for each scenario analyzed in Table 8 is the average of the results of running the simulation multiple times. In analyzed scenario Y1, when the JP weight ρ1 decreased to 0.8, the total number of employed people decreased, which was especially noticeable in the traditional sector. This indicates the importance of JP’s impact on traditional industries. In the Y2 scenario, increasing the weight of JP increased employment in traditional sectors, but this offset the impact on the services sector.

**Table 8:** Employment impact of changes in single potential weights.

Sensitivity Weight					Population (Employment) Change			
Scenario	ρ1	ρ2	ρ3	ρ4	Total	Tradition	Innovation	Service
Y1	0.8	1	1	1	1938	611	705	622
Y2	1.2	1	1	1	2374	929	779	667
Y3	1	0.8	1	1	2188	809	741	638
Y4	1	1.2	1	1	2293	870	763	659
Y5	1	1	0.8	1	2238	831	756	651
Y6	1	1	1.2	1	2264	853	761	650
Y7	1	1	1	0.8	2277	854	764	658
Y8	1	1	1	1.2	2247	839	757	651

In the Y3 scenario, when weight ρ2 of traditional industry potential decreased, employment in the traditional sector tended to decrease. Conversely, in the Y4 scenario, an increase in ρ2 increased employment in the traditional sector while also having a positive effect on employment in the innovative sector.

Scenarios Y5 and Y6 explored the impact of changes to the innovative industry potential MPinnos. A decrease in ρ3 in Y5 slightly reduced innovation employment, but an increase in ρ3 in Y6 caused this employment to rise again. This confirms that innovation sector employment is sensitive to the weights of MPinnos.

In the Y7 scenario, the decline in weight ρ4 of the service industry potential led to a decrease in the total number of employed people, despite an increase in employment in the traditional sector. Finally, in the Y8 scenario, an increase in ρ4 led to increased employment in the service sector but did not lead to significant changes in overall employment patterns. This shows that weight adjustments of different potentials interact and can lead to complex results.

In this study, we synthesized the results of the sensitivity analysis derived from urban economic simulations to analyze the multifaceted impact of changes in the relative weights of JP and MPtras, MPinnos, MPservice on employment patterns. This provides an essential basis for understanding how the coordination of potential indicators can have a complex impact on different sectors of economic growth in the formulation of city economic policies and plans.

In the Table 9, scenarios Z1 and Z2 show a clear increase in employment in traditional and innovative sectors due to the increase in the weight of the JP and the traditional and innovative industry potential, respectively. Z3 shows that the weighting of JP and the service industry potential led to increased employment in the service sector. Z4 and Z5 indicate that the increase in the potential of traditional industries, innovative industries, and service industries had a positive effect on employment in the innovation and service sectors.

**Table 9:** Employment impact of changes in composite potential weights.

Sensitivity Weight					Population (Employment) Change			
Scenario	ρ1	ρ2	ρ3	ρ4	Total	Tradition	Innovation	Service
Z1	1.2	1.2	0.8	0.8	2392	938	786	668
Z2	1.2	0.8	1.2	0.8	2258	873	747	638
Z3	1.2	0.8	0.8	1.2	2335	917	761	658
Z4	0.8	1.2	1.2	0.8	1989	641	721	628
Z5	0.8	1.2	0.8	1.2	1982	620	725	636
Z6	0.8	0.8	1.2	1.2	1922	599	708	615
Z7	1.2	1.2	1.2	0.8	2351	934	765	652
Z8	1.2	1.2	0.8	1.2	2362	932	774	655
Z9	1.2	0.8	1.2	1.2	2419	964	787	668
Z10	0.8	1.2	1.2	1.2	1997	667	713	617

The Z6 scenario indicates that increasing the weight of innovative industry potential would have a limited impact on employment in the innovation sector, meaning that the impact of that potential on overall employment would be less than expected. Z7 indicates an increase in employment in traditional and innovative sectors and a decrease in service industry potential; however, it also indicates a decrease in the overall employed population, suggesting that diseconomy related to congestion may be a possible cause.

Z8 seeks to balance the positive impact of increasing the weight of traditional industry potential on urban employment with the negative externalities of congestion. In Z9, increasing the weight of innovation and the service industry potential had a positive effect on overall employment but suggests a need to be wary of externalities due to congestion.

Finally, in the Z10 scenario, despite the reduction in the weight of the JP, the total number of employed people decreased due to the increase in the weight of other potentials. This indicates that additional economic activity can lead to negative outcomes by causing congestion; it suggests that the impact of diseconomy, which hinders synergies between industrial sectors, should be considered.

To summarize, the Table 8 shows the change in employed population across the traditional, innovative, and service sectors of the city’s economy, with each change in their potential weights. These results suggest that the JP primarily affects traditional sectors, while the Marshall potential has the capacity to influence those sectors and the economy more broadly. The Table 9 extends this analysis further, showing results for scenarios involving multiple weight changes. This table reveals the complex impact of weight adjustments, especially in the services sector, and shows how increasing certain weights can augment or offset the effects of other potentials.

The results of this sensitivity analysis provide policymakers with an evidence-based approach to fine-tuning urban economic models to understand the impact of adjusting potential indicators on overall employment and employment by economic sector. When formulating economic policies and urban planning, the results of this study will help policymakers prepare more effective strategic responses by clearly explaining the complex impact of potential adjustments on various sectors of economic growth.

Ultimately, these results highlight the importance of fine-tuning urban economic policies and planning, showing that it is essential to deeply understand and evaluate the impact of changes in potential weights on each economic sector. Additionally, by assessing the sensitivity of each potential indicator, a more cautious approach is required when designing policies for urban economic growth and sustainability.

## Discussion

This study examined the intricate interplay between agglomeration and congestion diseconomies, revealing the nuanced interactions that underlie urban dynamics. Focusing on how the variations in congestion parameter *λ* influence the evolution of urban patterns, we systematically analyzed the balance between clustering and sprawl within the development process. Notably, the transition between aggregation and dispersion observed at the threshold values of the congestion parameter offers profound insights that deepen our understanding of the interdependence between urban planning and economic dynamics.

The oscillation wave framework adopted in our study effectively depicted the delicate organization of intersectoral dynamic interactions, enabling a micro-level understanding of how specific economic sectors, including innovative industries, respond to urban agglomeration and congestion. This provides profound insights into the adaptability and resilience of each economic sector, laying a robust foundation for applying theoretical frameworks to urban planning and policy formulation.

Furthermore, our study has the potential to make significant contributions to the field of population dynamics. Analyzing the impact of economic activity and congestion levels on population distribution and mobility offers a fresh perspective on demographic dynamics. This understanding provides essential insights into phenomena related to urbanization, such as migration, fertility rate changes, and aging, which are crucial for establishing population policies and planning.

The Oscillation and Wave Framework developed in this study makes meaningful contributions to simulating long-term urban dynamics in a changing urban environment and evaluating the effectiveness of urban planning strategies. However, for academic precision and to further increase the depth of this research, it is essential to consider the study limitations.

First, this study’s approach does not provide a complete solution to a wide range of urban problems and complexities. Real-world cities are formed by the complex interaction of various actors, such as household economic conditions, changes in transportation infrastructure and land use, developers’ economic decisions, and policymakers’ political influence. Current models simplify or capture only a portion of the specific behavioral patterns and strategic interactions of these actors, having limitations in fully reflecting the multidimensionality of the overall urban ecosystem.

Additionally, the framework presented in the study does not sufficiently reflect the influence of all actors needed to explain and predict a city’s overall economic situation in the urban planning and economic policy-making process. The model focuses on individual actors or groups, such as the migration decisions of households or the investment patterns of developers, or explains how these individual factors interact and determine the macro-structure and long-term trends of the city’s economy. It does not provide a complete explanation of what is forming.

Finally, the proposed framework has not been thoroughly tested in complex real-world case studies. The current case study was conducted based on carefully selected variables and conditions; however, it is also influenced by the interaction of various actors including the economic diversity of households, detailed behavior of transportation and land use, and complex strategic decisions by developers and policymakers. The applicability and validity of the model in more complex urban environments is yet to be fully tested.

Efforts to clearly recognize and overcome these limitations will play an important role in providing directions for future research, adding depth to research, and deepening our understanding of urban modeling and policymaking processes. These efforts will serve as an opportunity to promote the development of theoretical and practical approaches to gain insight into complex urban problems and effectively respond to them in academia and practice.
